# Design development and optimisation of multifunctional Doxorubicin-loaded Indocynanine Green proniosomal gel derived niosomes for tumour management

**DOI:** 10.1038/s41598-023-28891-8

**Published:** 2023-01-30

**Authors:** Jaison Darson, Radha Thirunellai Seshadri, Kajal Katariya, Mothilal Mohan, Manjunath Srinivas Kamath, Meher Abhinav Etyala, Gopalakrishnan Chandrasekaran

**Affiliations:** 1 Department of Pharmaceutics, SRM College of Pharmacy, Kattankulathur, 603 203 India; 2 Department of Biotechnology, Faculty of Science and Humanities, SRM Institute of Science and Technology, Kattankulathur, 603 203 India; 3 Centre for Nanoscience and Nanotechnology, Sathyabama Institute of Science and Technology, Jeppair Nagar, Chennai, 600119 Tamil Nadu India; 4 K G Reddy College of Engineering and Technology, Moinabad, 500059 Telangana India; 5 Department of Physics and Nanotechnology, SRM Institute of Science and Technology, Kattankulathur, 603 203 India

**Keywords:** Cancer, Chemical biology

## Abstract

This study presents the design, development, and optimization of multifunctional Doxorubicin (Dox)-loaded Indocyanine Green (ICG) proniosomal gel-derived niosomes, using Design of Experiments (2^3^ factorial model). Herein, the multifunctional proniosomal gel was prepared using the coacervation phase separation technique, which on hydration forms niosomes. The effect of formulation variables on various responses including Zeta potential, Vesicle size, entrapment efficiency of Dox, entrapment efficiency of ICG, Invitro drug release at 72nd hour, and NIR hyperthermia temperature were studied using statistical models. On the basis of the high desirability factor, optimized formulation variables were identified and validated with the experimental results. Further, the chemical nature, vesicle morphology, surface charge, and vesicle size of optimized proniosomal gel-derived niosomes were evaluated. In addition, the effect of free ICG and bound ICG on NIR hyperthermia efficiency has been investigated to demonstrate the heating rate and stability of ICG in the aqueous environment and increased temperature conditions. The drug release and kinetic studies revealed a controlled biphasic release profile with complex mechanisms of drug transport for optimized proniosomal gel-derived niosomes. The potential cytotoxic effect of the optimised formulation was also demonstrated invitro using HeLa cell lines.

## Introduction

Over several decades, delivering the drugs directly into the target tissues without harming the neighboring tissues has been the biggest challenge in the treatment of cancer. Targeted drug delivery is an approach that is especially attempted to overcome these challenges^1–5^. Among various methods, vesicular drug delivery systems have gained special attention due to their capability to (1) enhance bioavailability and stability of the drug, (2) encapsulate both hydrophilic and hydrophobic drug, (3) prolong the circulation lifetime, and (4) achieve targeted delivery of drugs while minimizing the toxicities. In general, niosomal approach was applied to have a sustained release of low therapeutic index and low water solubility drugs^6^. Despite their advantages, vesicular systems such as liposomes and niosomes suffer from physical instability and premature leakage of drugs^7–9^. These limitations pertaining to niosomes and liposomes can be addressed by a proniosomal approach due to their stability and non-aggregation behavior during storage^10^.

Proniosomes gels are liquid crystalline gels composed of a mixture of surfactants, cholesterol, and lecithin. These are often lamellar and closed bilayer vesicles in structure, which on hydration form niosomes. The presence of hydrophilic, amphiphilic, and lipophilic moieties in the structure aids the incorporation of drug molecules in it. In general, proniosomes behave similar to liposomes in-vivo, possessing high metabolic stability and prolonged circulation time. The parenteral proniosomal preparations are found to be advantageous over conventional parenteral preparations or constant intravenous infusions in achieving patient compliance, as they can offer constant plasma concentration, improved efficacy, and reduced toxicity^11,12^. Proniosomes are highly stable physically and can be easily stored, distributed, sterilized, and separated into unit doses for parenteral delivery^13^.

Recently, a multifunctional approach including various treatment strategies with imaging modality has gained a special focus due to their capability to track the treatment progress in-vivo. In several works, NIR dye Indocyanine green (ICG) is used as a fluorescent imaging agent due to its high contrasting ability and high sensitivity. ICG is also an ideal photosensitizer for laser mediated photo-thermal therapy to elevate the local temperature by absorbing the laser energy around ⁓ 800 nm^14,15^. The dual-functional probe of ICG with integrated photothermal and optical imaging capability has attracted this NIR dye for the present work^16^. In this work, an anthracycline derivative Doxorubicin (Dox) was chosen as a model chemotherapeutic drug, owing to its narrow therapeutic index, low solubility, and increased risk of toxicity with higher peak plasma levels^17^. The aim of the current work is to incorporate Indocyanine Green and Doxorubicin into proniosomes in order to achieve a combined effect of chemotherapy and photothermal therapy, while increasing the therapeutic index of doxorubicin. In this study, 2^3^ factorial experiments were adopted to study the effect of cholesterol, egg lecithin, and span 60 content on various responses, including zeta potential (Y_1_), vesicle size (Y_2_), entrapment efficiency of Dox (Y_3_), entrapment efficiency of ICG (Y_4_), Invitro drug release at 72nd hour (Y_5_) and NIR hyperthermia temperature (Y_6_) by using design of experiments (DoE) Design-Expert version 12.0.5.0 (Stat-Ease. Inc, Minneapolis, MN)^18^.

## Materials and methods

### Chemicals used

Doxorubicin Hydrochloride was obtained as a gift sample from NATCO Pharma Ltd., Chennai. Indocyanine Green, MP biomedicals, LLC, France, Span 60 (W/O emulsifier), Egg Lecithin (emulsifying and stabilizing agents), and Cholesterol (emulsifying agent and they can readily absorb water to form stable emulsions), SRL, was obtained from SISCON, Chennai. Dialysis Membrane was obtained from Himedia Laboratories, Mumbai. All the reagents and chemical substances used were of analytical and pharmaceutical category.

### Experimental design

2^3^ factorial design was adopted based on the preliminary experiments to screen and identify the optimized Dox-loaded ICG proniosomal gel-derived niosomes. The concentration of Span 60 (A), Cholesterol (B), and Egg Lecithin (C) were chosen to study their effect on zeta potential (Y_1_), vesicle size (Y_2_), entrapment efficiency of Dox (Y_3_), entrapment efficiency of ICG (Y_4_), Invitro drug release at 72nd hour (Y_5_) and NIR hyperthermia temperature (Y_6_). The factors were studied at two levels, 0 and 1, indicating the low and high levels, and the optimized formulation was accomplished with the help of the design of experiments (DoE), Design-Expert version 12.0.5.0. The actual and coded levels of variables are represented in Table 1, and the experimental design of 2^3^ factorial is presented in Table 2.

**Table 1:** Actual and coded levels of variables.

Variable		Low level	High level
	Coded value	− 1	1
Amount of span 60 (A) (μM)		7.5	15.0
Amount of cholesterol (B) (μM)		6.0	8.0
Amount of egg lecithin (C) (μM)		6.0	12.0

**Table 2:** Experimental design of 2^3^ factorial design.

Run	Span 60 (A)	Cholesterol (B)	Egg lecithin (C)	Dox	ICG
	μM	μM	μM	mg	mg
1	15	6.0	6.0	10.0	1.0
2	7.5	6.0	6.0	10.0	1.0
3	15	8.0	12.0	10.0	1.0
4	15	8.0	6.0	10.0	1.0
5	7.5	8.0	12.0	10.0	1.0
6	7.5	8.0	6.0	10.0	1.0
7	7.5	6.0	12.0	10.0	1.0
8	15.0	6.0	12.0	10.0	1.0

### Preparation of Dox-loaded ICG proniosomal gel-derived niosomes

The preparation of Dox-loaded ICG proniosomal gel was carried out by using a coacervation-phase separation process. Varying concentrations of span 60, cholesterol, and egg lecithin were transferred into 15 mL glass bottles. 3 mL of ethanol was added to previously weighed proniosomal contents, kept at a temperature of about 60 °C with constant stirring at 500 rpm, until a clear solution was obtained. To this clear solution, 10 mg of Doxorubicin and 1 mg of Indocyanine Green previously dissolved in methanol (2 mL) was transferred and stirred for 30 min to obtain a homogenous mixture, cooled to room temperature. 5 mL of an aqueous phase (deionized water) was introduced into the drug-surfactant mixture under probe sonication for 5 min. The coacervate obtained was kept aside overnight to obtain proniosomal gel^19^. When needed, the proniosomal gel is reconstituted with phosphate buffer (pH 7.4) using a vortex shaker to form niosomes.

### Characterization of Dox-loaded ICG proniosomal gel-derived niosomes

#### Chemical interaction studies

The chemical interactivity between actives and proniosomal contents were investigated from the Fourier Transform Infra-Red Spectrum recorded by using IR Tracer 100 AH (using ATR accessory), Shimadzu, Japan recorded from 4000 to 400/cm under ambient temperature conditions.

#### Vesicle morphology

The vesicle morphology of prepared proniosomes was studied by using FESEM. The sample for FESEM studies was prepared by drop-casting the niosomal dispersion over the aluminium substrate, followed by lyophilization. The Transmission Electron Microscopic (TEM) images of niosomal dispersion coated on the copper grid were recorded at 200 kV using JEM 2100 plus, JOEL, Japan to analyze the vesicular formation after hydration.

#### Vesicle size

The average vesicle size of niosomal dispersion was determined by measuring the Brownian motion using Malvern Zetasizer (Dynamic Light Scattering technique), at room temperature by keeping the scattering detector angle at 90°. The niosomal dispersion employed for the measurement of vesicle size was derived by dispersing Dox-loaded ICG proniosomal gel in aqueous media using a vortex shaker.

#### Zeta potential

The zeta potential values of niosomal dispersion were obtained by using Malvern Zetasizer (Electrophoretic Light Scattering technique). The niosomal dispersion employed for the measurement of zeta potential was also derived by dispersing Dox-loaded ICG proniosomal gel in aqueous media using a vortex shaker.

#### Entrapment efficiency

The percentage entrapment efficiency (EE) of Dox-loaded ICG proniosomal gel-derived niosomes were estimated by measuring the unentrapped Dox and ICG present in the supernatant liquid^20^ by using UV–Vis-NIR spectrophotometer, Shimadzu at λmax of about 480 nm^21^ and 808 nm^22^, respectively. The percentage entrapment efficiency (EE) was calculated using the Eq. (1),

1 $$EE\left( \% \right) = \frac{Total \;amount\; of\; Dox\; or \;ICG - Unentrapped\; Dox \;or \;ICG}{{Total\; amount \;of\; Dox \;or \;ICG}} \times 100$$

#### NIR-hyperthermia

Dox-loaded ICG proniosomal gel-derived niosomes equivalent to 0.01 mg of Indocyanine green were weighed and dispersed in 2 mL of phosphate buffer pH 7.4. The so-formed niosomal dispersion was irradiated with the Infrared light at 808 nm in the UV–Vis-NIR spectrophotometer. The change in temperature of niosomal dispersion with respect to time was recorded using Neoptix T1™ optical temperature sensor, Qualitrol Corp. The maximum hyperthermia potential of Dox-loaded ICG proniosomal gel-derived niosomes was determined from the data obtained.

#### Percentage drug release studies and drug release kinetics

In vitro drug release studies were carried out for both pure drug doxorubicin and Dox-loaded ICG proniosomal gel-derived niosomes (equivalent to 2 mg of Doxorubicin) in 5 mL of phosphate buffer pH 7.4 in a dialysis bag (10 kDa cut off) and immersed into 50 mL of phosphate buffer pH 7.4. The media was maintained at 37 °C ± 0.5 °C and agitated at 50 rpm using a magnetic stirrer. Periodically, 2 mL of the sample was pipetted out and replaced with 2 mL of phosphate buffer pH 7.4. The percentage drug release was estimated by using UV–Vis-NIR Spectrophotometer at 480 nm. Similarly, the drug release study was performed for both pure drug doxorubicin and Dox-loaded ICG proniosomal gel-derived niosomes by replacing phosphate buffer pH 7.4 dissolution medium with 0.1 M acetate buffer pH 5.5. The sink conditions were maintained throughout the release study by using a volume of dissolution media more than the saturation solubility^23,24^ of doxorubicin hydrochloride. The in-vitro drug release kinetics of Dox-loaded ICG proniosomal gel-derived niosomes were studied using various mathematical models such as Zero order, First order, Noyes-Whiney model, Korsmeyer-Peppas, Hixson-Crowell, Higuchi diffusion kinetics, and Weibull models^25^.

#### Statistical analysis

The relationship between formulation variables and responses was studied by employing two factorial designs. The mean values of experimental data were fitted in the following polynomial equations,

2 $$Y = \beta_{0} + \beta_{1} X_{1} + \beta_{2} X_{2} + \beta_{3} X_{3} + {\upvarepsilon }$$

3 $$Y = \beta_{0} + \beta_{1} X_{1} + \beta_{2} X_{2} + \beta_{3} X_{3} + \beta_{4} X_{1} X_{2} + \beta_{5} X_{1} X_{3} + \beta_{6} X_{2} X_{3}$$

where Y represents the predicted responses (zeta potential (Y_1_), vesicle size (Y_2_), entrapment efficiency of DOX (Y_3_), entrapment efficiency of ICG (Y_4_), Invitro drug release at 72nd hour (Y_5_), and NIR hyperthermia temperature (Y_6_)), ${\beta }_{0}$ is the arithmetic mean of responses (intercept), ${\beta }_{1},{\beta }_{2}{, \beta }_{3}$ are the regression coefficients, $\upvarepsilon$ is the model residual, ${X}_{1},{X}_{2},{X}_{3}$ corresponds to independent variables, and ${X}_{1}{X}_{2},{ X}_{1}{X}_{3},{ X}_{2}{X}_{3}$ corresponds to the two way interaction effects. ANOVA was carried out to ascertain the statistical significance (p ≤ 0.05) of each coefficient term. Similarly, a test for lack of fit was performed on the suggested model with a 95% confidence interval to check how well the statistical model fit. The regression coefficient (R^2^) and Adjusted R^2^ were established to evaluate the goodness of fit of experimental data.

#### Optimization and validation

Based on the maximum desirability function, optimized formulation variables for the preparation of Dox-loaded ICG proniosomal gel with high zeta potential (> ± 30), maximum entrapment efficiency (%), lesser vesicle size, controlled release of drug (%), and maximum NIR hyperthermia temperature were preferred. The accuracy and suitability of optimized formulation variables was validated by performing experiments in triplicate, and their mean was compared with the predicted value.

#### Invitro anti-cancer activity

The in-vitro anti-cancer activity were carried out using HeLa cells cultured in DMEM/F-12 medium supplemented with 1% penicillin, 1% streptomycin, and FBS (10% v/v), and incubated at 37 °C with 5% CO_2_. The grown cells were harvested by trypsinization (0.03% w/v EDTA solution and 0.25% w/v trypsin), and about 1 × 10^4^ cells were seeded in wells of a 12-well plate and incubated for 6 h for cell attachment. After cell attachment, the wells were treated with 500 µg/mL of bare proniosomal gel (negative control—without Dox and ICG), 5 µg/mL of doxorubicin hydrochloride (positive control), 50 µg/mL, 100 µg/mL, 200 µg/mL, 300 µg/mL and 500 µg/mL of Dox-loaded ICG proniosomal gel-derived niosomes (DIP) for 24 h along with a negative control (without treatment). The media was detached after treatment by washing with phosphate buffer pH 7.4. 200 µL of MTT (4 mg/mL) was added to the wells under a dark environment and incubated for 4 h at 37 °C with 5% CO_2_. After the incubation period, MTT was carefully removed using 200 µL of DMSO. The cell viability was estimated by reading the extent of formazan formation in each well with the help of an ELISA plate reader at 560 nm^26^. The cell viability was determined using the following formula (Eq. 4),

4 $$Cell\; Viability \left( \% \right) = \frac{{Absorbance\; \left( {Test} \right)}}{{Absorbance \;\left( {Negative \;Control} \right)}} \times 100$$

Data were expressed as mean (${\overline{\text{x}}}$) ± standard deviation (SD).

## Results and discussion

### Chemical interaction studies

The chemical nature and compatibility of Doxorubicin, Indocyanine Green with other proniosomal contents such as Span 60, Cholesterol, and Egg lecithin were studied from their vibrational frequencies obtained using FTIR spectroscopy.

**Figure 1:**
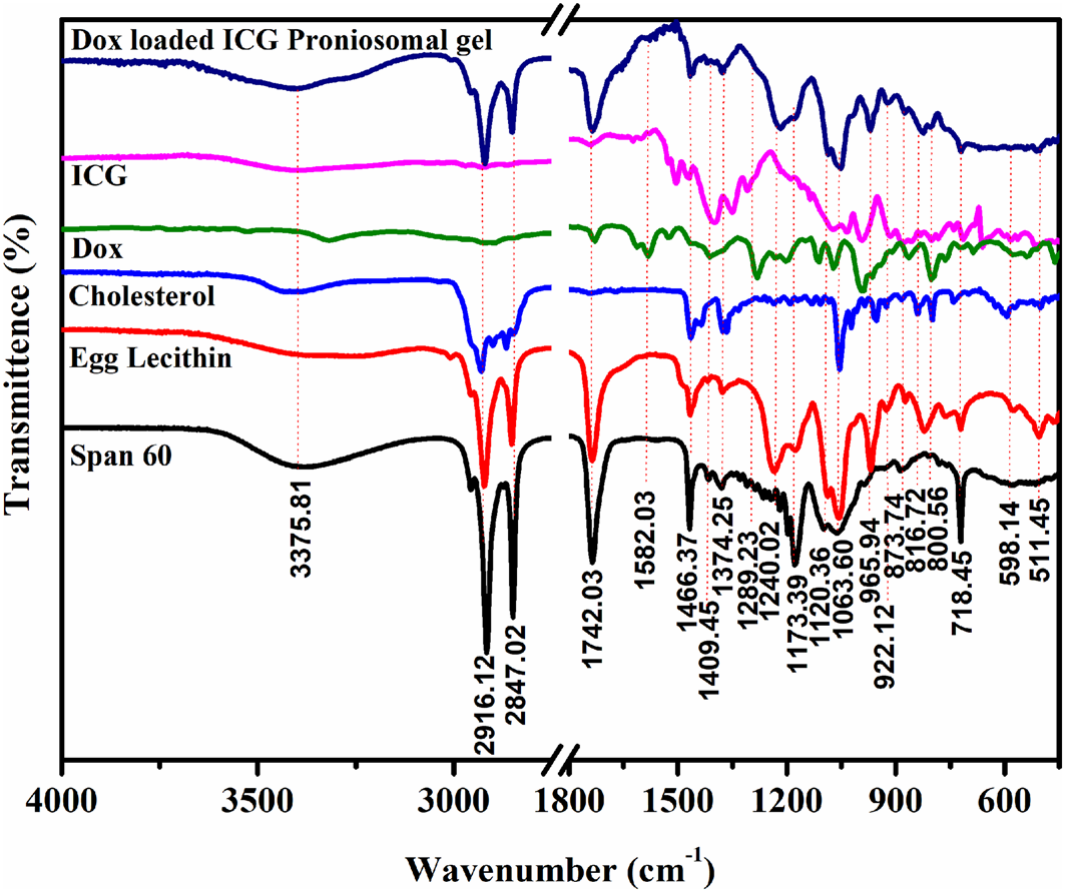
Fourier Transform Infra-Red Analysis of proniosomal contents.

Figure 1 shows strong characteristic band of νO-H (3375.81/cm), νC-H Stretching of Methyl and Methylene (2916.12/cm and 2847.02/cm), νC=O stretching (1742.03/cm), νC-O stretching (1240.02/cm) and νC–O–C stretching (1063.60/cm) corresponding to Span 60, Cholesterol and Lecithin. Doxorubicin gives characteristic bands at 3322.18/cm (νO-H), 1724.97/cm (νC=O), 1283.94/cm (νC-O), 1117.41/cm (νC-O-C) and 1069.71/cm (νC-N)^27^. ICG gives characteristic bands at 3414.71/cm (νO-H), 1748.85/cm (νC=O), 1617.20/cm (aromatic ring), 1510.75/cm (νC=C), 1408.57/cm (νC-H), 1343.02/cm (defC-H), 1304.19/cm (νC-O), 1283.94/cm (νC-O), 1117.41/cm (νC-O-C) and 1075.36/cm (νS = O). Dox-loaded ICG proniosomal gel gives a characteristic band for the functional groups of Span 60, Cholesterol, and Egg lecithin with a slight shift in the wavenumber, indicating the absence of chemical interaction between the excipients, ICG, and Doxorubicin. The absence of drug and ICG related bands indicates the effective encapsulation of drug inside the bilayer membrane of proniosomes.

### Pareto analysis and statistical models

Pareto principle (t-value) utilize the classic 80/20 rule to give the information needed to prioritize the effort in identifying the most positive impact of factors based on the frequency of occurrence, whereas statistical significance (p-value) gives the confidence that the change in selected factors has an impact on responses^28^. The responses such as zeta potential, vesicles size, entrapment efficiency of DOX, entrapment efficiency of ICG, in-vitro drug release at 72nd hour, and NIR hyperthermia temperature observed through experimental studies for the formulation variables are presented in Table 3. The statistical significance of model terms is represented in Table 4. The interactive effects and model diagnostic plots of formulation variables on responses are presented in the supplementary information.

**Table 3:** Responses of Dox-loaded ICG proniosomal gel-derived niosomes.

Run	A	B	C	Zeta potential (Y_1_)	Vesicle size (Y_2_)	Entrapment efficiency of Dox (Y_3_)	Entrapment efficiency of ICG (Y_4_)	Invitro Drug release at 72nd hr (Y_5_)	NIR Hyperthermia temperature (Y_6_)
				mV	nm	%	%	%	ºC
1	1	− 1	− 1	− 29.12 ± 1.51	195.8 ± 3.9	85.37 ± 1.51	95.74 ± 1.09	47.28 ± 3.56	57.1 ± 1.1
2	− 1	− 1	− 1	− 25.45 ± 2.11	169.7 ± 2.8	78.42 ± 0.89	88.45 ± 1.34	57.12 ± 2.11	53.6 ± 1.8
3	1	1	1	− 32.54 ± 0.89	276 ± 7.2	52.21 ± 0.91	77.88 ± 2.97	21.01 ± 1.99	47.6 ± 0.6
4	1	1	− 1	− 26.16 ± 2.09	212.6 ± 3.2	68.54 ± 2.21	82.95 ± 1.22	31.1 ± 1.34	51.2 ± 0.8
5	− 1	1	1	− 31.65 ± 1.71	239.6 ± 5.5	49.82 ± 0.82	73.21 ± 1.67	24.69 ± 1.67	48.6 ± 1.2
6	− 1	1	− 1	− 24.56 ± 0.91	185.1 ± 3.4	63.59 ± 1.13	78.84 ± 1.91	44.57 ± 2.11	51.5 ± 1.5
7	− 1	− 1	1	− 32.25 ± 1.23	225.4 ± 3.8	74.69 ± 2.91	83.45 ± 2.05	49.22 ± 1.09	53.8 ± 0.7
8	1	− 1	1	− 35.12 ± 1.49	255.0 ± 6.2	79.56 ± 3.01	91.54 ± 2.22	50.23 ± 1.76	56.2 ± 0.6

Data represented as the mean ± SD (n = 3).

**Table 4:** Descriptive statistical analysis of models.

Responses	p-values of factors							Fit statistics				
	Model	A	B	C	AB	AC	BC	Adjusted regression coefficient (${R}_{a}^{2}$)	Predicted regression coefficient (${R}_{p}^{2}$)	Regression coefficient (${R}^{2}$)	Coefficient of variance (% CV)	Adequate precision
Zeta potential (mV)	0.0058*	0.0063*	0.0081*	0.0022*	0.0141*	0.0379*	0.0850	0.9999	0.9994	1.0000	0.1075	355.5386
Vesicle size (nm)	< 0.0001*	0.0001*	0.0011*	< 0.0001*	–	–	–	0.9938	0.9859	0.9965	1.29	52.1541
Entrapment efficiency of Dox (%)	0.0093*	0.0159*	0.0036*	0.0077*	0.0680	0.0656	0.0149*	0.9998	0.9984	0.9999	0.246	224.6999
Entrapment efficiency of ICG (%)	0.0080*	0.0063*	0.0033*	0.0077*	0.0231*	0.1112	0.1010	0.9988	0.9988	1.0000	0.101	284.601
Invitro drug release at 72nd hour (%)	0.0201*	0.0260*	0.0082*	0.0193*	0.0807	0.0327*	0.0270*	0.9992	0.9927	0.9999	0.9219	102.2501
Hyperthermia temperature (ºC)	0.0297*	0.0552	0.0117*	0.0353*	0.0353*	0.1392	0.0438*	0.9982	0.9839	0.9997	0.2696	71.8132

*Significant p value (< 0.05), model term p value (< 0.10).

The adequate precisions ratio of various formulations was found to be higher than 4, indicating the desirability of the model for design space. The predicted regression coefficients of the model were relatively closer to unity, indicating their goodness of fit. Also, the regression coefficients ${R}_{p}^{2}$ and ${R}_{a}^{2}$ were relatively very close, indicating the reasonable agreement between predicted and adjusted regression coefficients. The interactive model was chosen for the responses zeta potential (Y_1_), entrapment efficiency of Dox (Y_3_) and ICG (Y_4_), Invitro drug release at 72nd hour (Y_5_), and NIR hyperthermia temperature (Y_6_), as it exhibited better descriptive statistics. However, in the case of vesicle size, the linear order model was observed to have a better mathematical and statistical descriptive.

#### Effect of formulation variables on zeta potential

**Figure 2:**
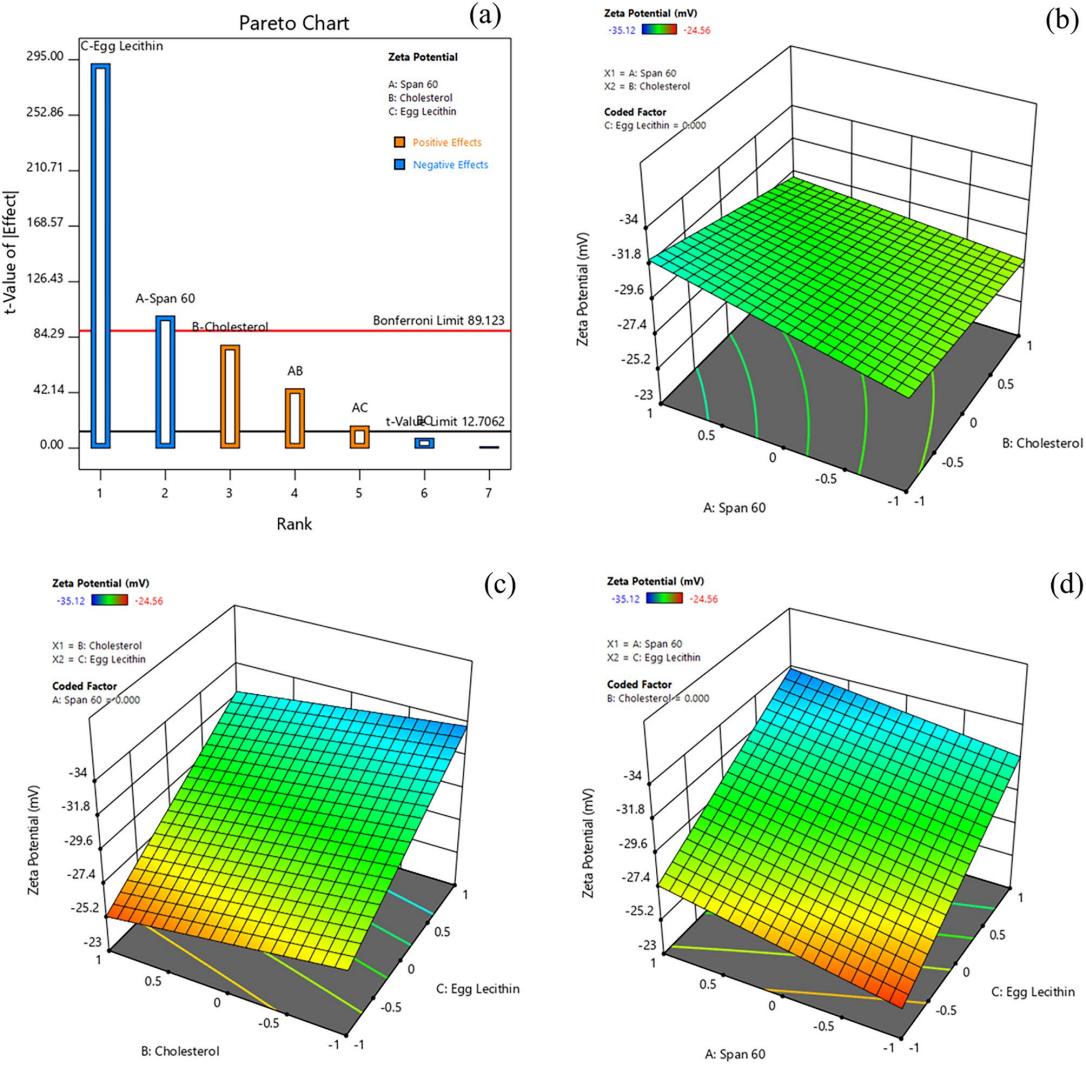
Effect of formulation variables (**a**) Pareto chart of zeta potential, (**b**) Span60*Cholesterol on zeta potential, (**c**) Cholesterol* Egg lecithin on zeta potential, (**d**) Span60*Egg Lecithin on zeta potential [Design-Expert version 12.0.5.0].

The zeta potential (≤ ± 30 mV) is considered as a critical parameter to maintain the suspension stability of nano-sized particles. The zeta potential determined for various formulations ranged from − 35.12 to − 24.56 mV (Table 3). The Pareto chart and 3D-response surface plots (Design-Expert version 12.0.5.0) indicating the effect of formulation variables on zeta potential values are presented in Fig. 2a–d. Table 4 indicates that all factors (A, B, C, AB, and AC) except BC had a significant effect on the zeta potential of Dox-loaded ICG proniosomal gels. The interactive polynomial equation of Y_1_ with coded factors derived from the responses is shown in Eq. (5).

5 $$Y_{1} = - 29.6063 - 1.12875 A + 0.87875 B - 3.28375 C + 0.50625 AB + 0.18875 AC - 0.08375 BC$$

From Eq. (5) and Pareto chart (Fig. 2a), it was observed that egg lecithin (C) displayed the strongest negative effect on the zeta potential of vesicles. The increase in the concentration of egg lecithin promotes negative zeta potential of proniosomal vesicles due to the domination of negatively charged phosphate moieties of zwitterionic egg lecithin, favored at neutral pH^29^. Furthermore, span 60 (A) also showed a significant negative effect on zeta potential, promoting negative surface potential of proniosomal vesicles due to their preferential surface adsorption of hydroxyl ions from the aqueous phase^30^. It was also observed that cholesterol (C) demonstrated an inverse (or positive) effect on zeta potential. Though the role of amphipathic cholesterol was not understood completely, several reports highlighted that increased cholesterol content favors their insertion into bilayer vesicles. The cholesterol occupied within the vesicles reduces repulsive forces of like-charges while increasing their hydrophobicity. This increase in hydrophobicity decreases the average surface charge density of vesicles^30^.

The model also indicates that the interactive effect of span60 and cholesterol (AB), span60, and egg lecithin (AC) showed a significant positive effect on zeta potential. The decreasing negative potential of span60*cholesterol (AC) interaction could be related to the distortion of vesicular arrangements of non-ionic surfactants, which is further influenced by increasing hydrophobicity of proniosomal vesicles, exerted with an increase in cholesterol concentration. Also, the decreasing negative potential observed with span 60*egg lecithin interaction could be due to vesicular distortions influenced by amplification of electrostatic repulsive force between span 60 and egg lecithin head groups having a similar charge. The increased electrostatic repulsion within the vesicles could be due to the high charge density of anionic vesicles^30^.

#### Effect of formulation variables on vesicle size

**Figure 3:**
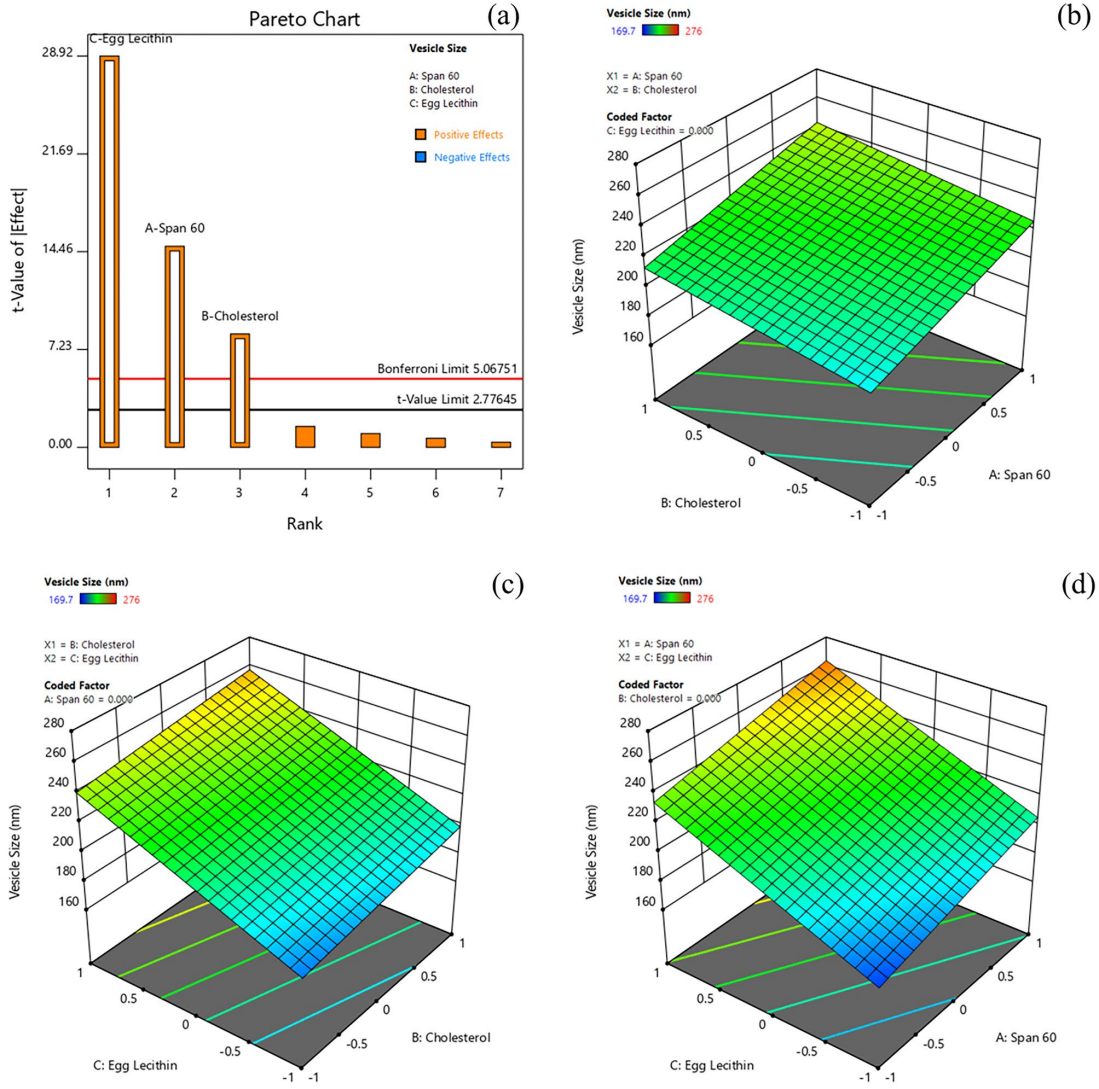
Effect of formulation variables (**a**) Pareto chart of vesicle size, (**b**) Span60*Cholesterol on vesicle size, (**c**) Cholesterol* Egg lecithin on vesicle size, (**d**) Span60*Egg Lecithin on vesicle size [Design-Expert version 12.0.5.0].

Vesicle size is one of the critical factors to achieve longer circulation time in-vivo, by evading the reticuloendothelial system. The vesicle size determined for various formulations ranged from 169.7 to 276 nm (Table 3). The Pareto chart and 3D-response surface plots (Design-Expert version 12.0.5.0) showing the effect of formulation variables on the vesicle size are presented in Fig. 3a–d. Table 4 indicates that all factors (egg lecithin > span 60 > cholesterol) exerted a significant positive effect on vesicle size of Dox-loaded ICG proniosomal gels. The linear polynomial equation of Y_2_ with coded factors derived from the responses is shown in Eq. (6).

6 $${Y}_{2}=219.90+14.95 A+8.42 B+29.10 C$$

From Eq. (6) and Pareto chart (Fig. 3a), it was observed that egg lecithin (C) displayed the strongest positive effect on vesicle size. The mean vesicle size was greatly increased with an increase in lecithin content. This could be possibly due to the long saturated (18C) and unsaturated (18C) hydrocarbon chain of egg lecithin molecules, which increases the bilayer thickness of proniosomal vesicles. Furthermore, the increased mean vesicle size with an increase in span 60 could have been resulted due to the formation of a large micellar structure^19^. The Eq. (5) also revealed a positive effect of cholesterol on mean vesicle size. It is expected that at low concentration, the cholesterol reduces the mean vesicle size due to close packing of surfactant monomers. Whilst, at high concentration, cholesterol enters the bilayer vesicle structure, increasing their chain order with hydroxyl group heading towards aqueous layer and aliphatic chain arranges themselves to the hydrophobic chains. This usually results in increased hydrophobicity of vesicles, which imparts a strain in the bilayer structure. Thus, to establish the thermodynamic stability of vesicles, the mean vesicle size is increased with high cholesterol content^19,30,31^.

#### Effect of formulation variables on entrapment efficiency of doxorubicin

**Figure 4:**
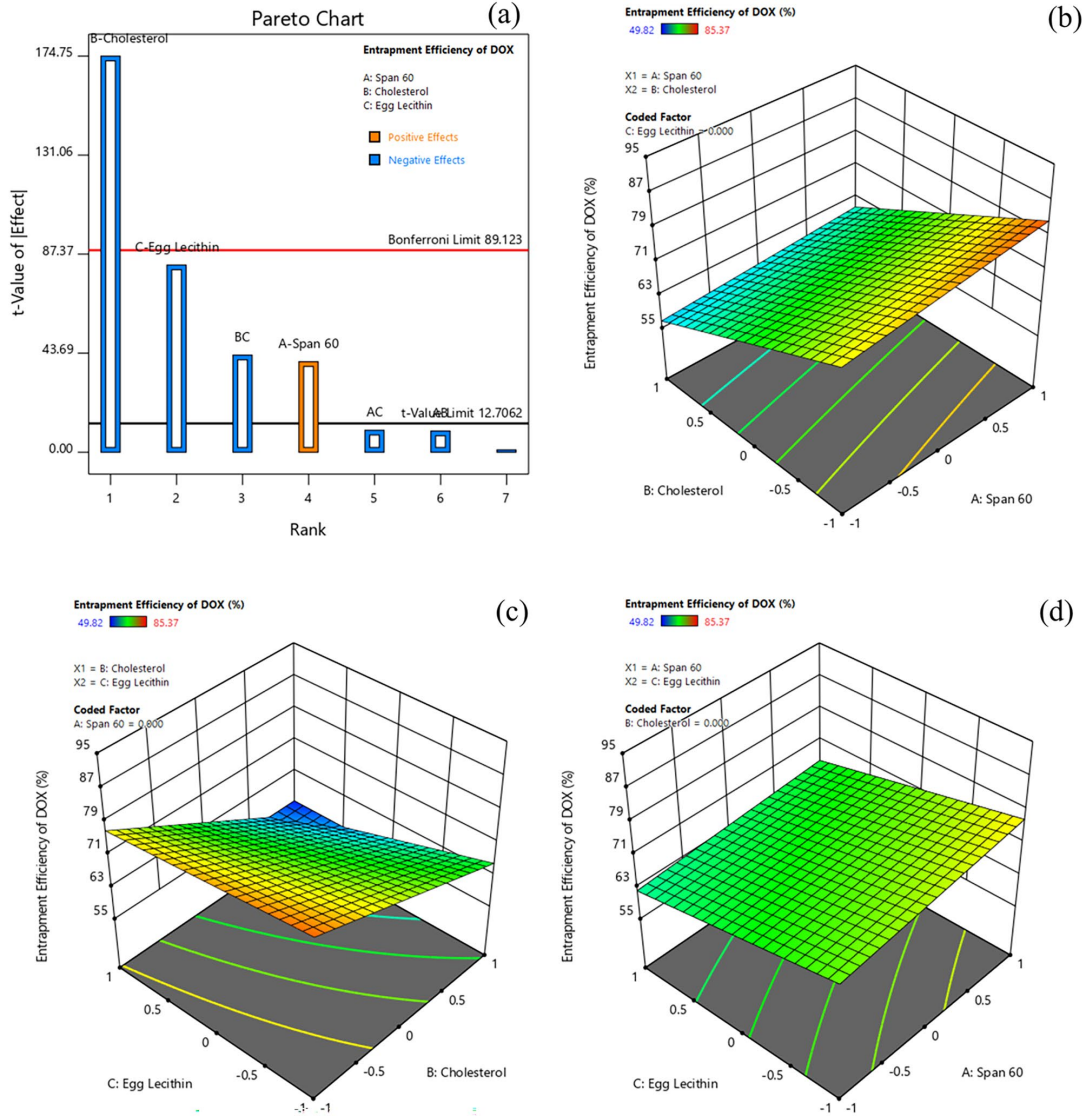
Effect of formulation variables (**a**) Pareto chart of Entrapment Efficiency (EE) of DOX, (**b**) Span60*Cholesterol on EE of DOX, (**c**) Cholesterol* Egg lecithin on EE of DOX, (**d**) Span60*Egg Lecithin on EE of DOX [Design-Expert version 12.0.5.0].

Entrapment of drug or other cargo molecules is considered the most pivotal factor in drug delivery carrier systems. The entrapment efficiency (%) of doxorubicin for various formulations ranged from 49.82 to 85.37% (Table 3). The Pareto chart and 3D-response surface plots (Design-Expert version 12.0.5.0) indicating the effect of formulation variables on entrapment efficiency of Dox are presented in Fig. 4a–d.

Table 4 indicates that all factors (A, B, C, and BC) except AB and AC had a significant effect on entrapment efficiency of doxorubicin in Dox-loaded ICG proniosomal gels. The interactive polynomial equation of Y_3_ with coded factors derived from the responses is shown in Eq. 7.

7 $${Y}_{3}=69.025+2.395 A-10.485 B-4.955 C-0.560 AB-0.580 AC-2.57 BC$$

From Eq. (7) and Pareto chart (Fig. 4a), it was observed that cholesterol (B) displayed a maximum negative effect on entrapment of doxorubicin. The increase in cholesterol concentration strongly affects the entrapment efficiency of doxorubicin, as both cholesterol (log P, + 7.02) and doxorubicin (log P, + 1.41) exhibits positive log P values (hydrophobic character). The positive partition coefficient values of cholesterol and doxorubicin indicate their higher affinity towards the hydrophobic layer. The increased tendency of cholesterol molecules (high log P) to occupy the spaces within the hydrophobic layer competes with the drug that needs to be entrapped; this leads to the expulsion of drugs from the bilayer vesicles^19,31–33^. The other possible reason behind the decreased entrapment efficiency could be due to the disruption of bilayer structure at higher cholesterol content^32^. Also, egg lecithin showed a significant negative effect on entrapment efficiency of doxorubicin due to their preferential uptake of cholesterol molecules that is highly lipophilic than doxorubicin^34^. The poor entrapment of doxorubicin in the egg lecithin-rich environment could also be due to the formation of mixed micelles with surfactants^35^. The interactive effect of cholesterol and egg lecithin (BC) was also observed to possess a significant negative effect, as the interaction of cholesterol with egg lecithin rigidifies the membrane and offers superior mechanical strength to the bilayer membrane, which hinders the entrapment of doxorubicin into the bilayer membrane. However, span 60 featured a significant positive effect on the drug entrapment and, their increased entrapment efficiency of doxorubicin with increased non-ionic surfactant content could be due to the formation of a larger number of proniosomal vesicles with an increasing dimension of hydrophobic bilayer^19,36^.

#### Effect of formulation variables on entrapment efficiency of Indocyanine Green

**Figure 5:**
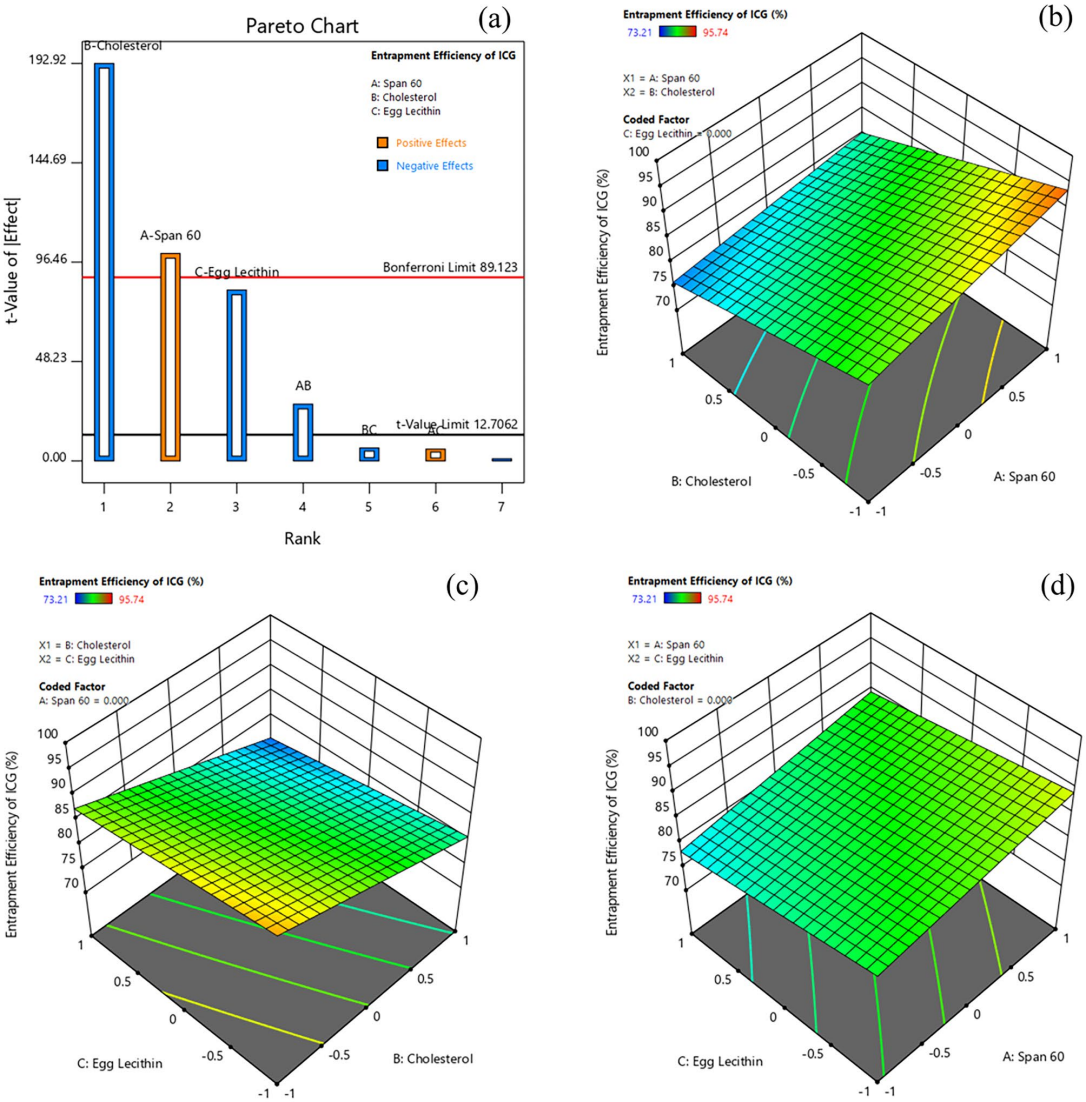
Effect of formulation variables (**a**) Pareto chart of Entrapment Efficiency (EE) of ICG, (**b**) Span60*Cholesterol on EE of ICG, (**c**) Cholesterol* Egg Lecithin on EE of ICG, (**d**) Span60*Egg Lecithin on EE of ICG [Design-Expert version 12.0.5.0].

The entrapment efficiency (%) of Indocyanine Green (ICG) for various formulations ranged from 73.21% to 95.74% (Table 3). The Pareto chart and 3D-response surface plots (Design-Expert version 12.0.5.0) indicating the effect of formulation variables on entrapment efficiency of ICG are presented in Fig. 5a–d. Table 4 indicates that all factors (A, B, C, and AB) except BC and AC had a significant effect on the entrapment efficiency of ICG in Dox-loaded ICG proniosomal gels. The interactive polynomial equation of Y_4_ with coded factors derived from the responses is shown in Eq. (8).

8 $${Y}_{4}=84.007+3.02 A-5.787 B-2.487 C-0.825 AB+0.17 AC-0.1875 BC$$

The Eq. (8) and Pareto chart (Fig. 5a) displayed the strongest negative effect of cholesterol on entrapment of ICG. The negative effect of cholesterol could be due to their higher affinity towards the hydrophobic layer, in comparison to the ICG (log P, + 4.17)^19,31^. Also, cholesterol at higher concentrations disrupts the bilayer structure, leading to the expulsion of ICG^32^. Furthermore, egg lecithin also displayed a significant negative effect on entrapment of ICG, as it can promote the formation of mixed micelles and preferentially uptake cholesterol molecules into the bilayer structure^35^. In contrary, span 60 featured a significant positive effect on the entrapment efficiency of ICG, which could be due to the formation of the increased number of vesicles with reduced hydrophobicity, allowing the accommodation of both lipophilic and hydrophilic drugs in larger fractions^36^. The interactive effect of span 60 and cholesterol (AB) revealed a significant negative effect on the entrapment of ICG. The increasing cholesterol concentration with decreased span 60 imparts an imbalance in the vesicular structure. In order to achieve a thermodynamically stable form with strengthened bilayer structures, the vesicle radius usually increases. This increase in bilayer rigidity diminishes their micro fluidity, leading to poor entrapment efficiency of ICG^37^.

#### Effect of formulation variables on drug release

**Figure 6:**
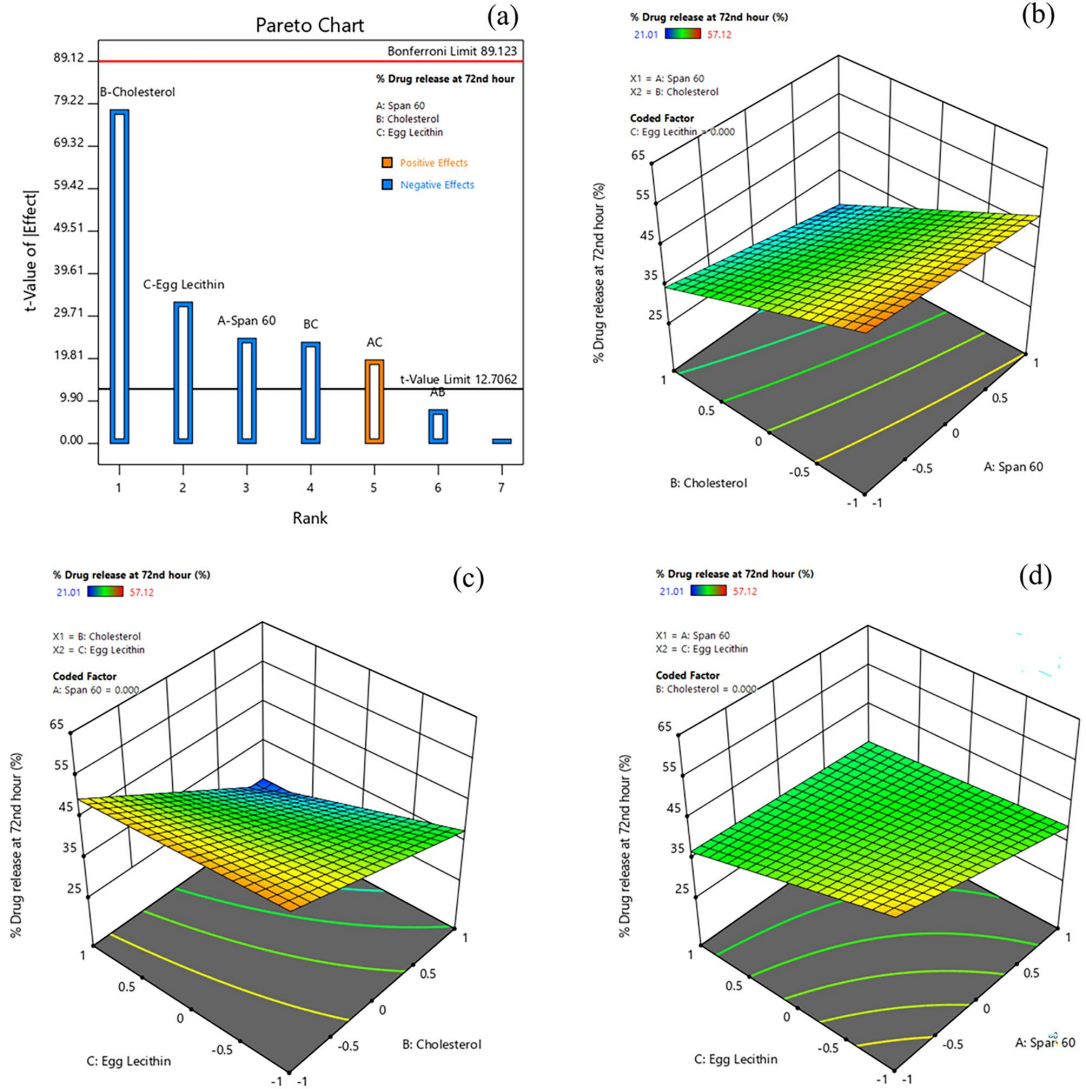
Effect of formulation variables (**a**) Pareto chart of Percentage Drug Release, (**b**) Span60*Cholesterol on Percentage Drug Release, (**c**) Cholesterol*Egg lecithin on Percentage Drug Release, (**d**) Span60*Egg Lecithin on Percentage Drug Release [Design-Expert version 12.0.5.0].

Percentage cumulative drug release is yet another important factor in the design of drug delivery systems, as controlled delivery of cargo molecules is a much-needed design to minimize drug-induced systemic toxicities and maximize the therapeutic benefit in cancer therapy. The percentage drug release of doxorubicin for various formulations ranged from 21.01 to 57.12% (Table 3) at the 72nd hour. The Pareto chart and 3D-response surface plots (Design-Expert version 12.0.5.0) indicating the effect of formulation variables on drug release (%) of doxorubicin are presented in Fig. 6a–d. Table 4 indicates that all factors (A, B, C, BC, and AC) except AB had a significant effect on the percentage drug release of doxorubicin from Dox-loaded ICG proniosomal gels. The interactive polynomial equation of Y_5_ with coded factors derived from the responses is shown in Eq. (9).

9 $${Y}_{5}=40.6525-3.2475 A-10.310 B-4.365 C-1.04 AB+2.58 AC-3.1275 BC$$

From Eq. (9) and Pareto chart (Fig. 6a), it was observed that Cholesterol (B) displayed the strongest negative effect on percentage drug release of doxorubicin from the proniosomal gel. The large negative effect of cholesterol indicates that increased cholesterol content decreased the percentage of drug release. In general, higher cholesterol content in proniosomal formulation not only stabilizes the membrane but also increases the hydrophobicity of vesicles. This increased hydrophobicity of vesicles decreases the permeability of the drug across the bilayer membrane, retarding the drug release from the proniosomal vesicles^36^. It was also found that egg lecithin played a significant effect in hindering the drug release, as lecithin at higher concentration decreases the thermodynamic activity of the drug, due to the formation of cylindrical micelles. These micelle structures formed have a tendency to trap the drug molecules within themselves, retarding their release. Also, the absorption of higher water content by lecithin into the core decreased the release rate of doxorubicin^38^. Furthermore, span 60 also pose a significant effect on retardation of drug release, as it has a similar tendency like egg lecithin to form micellar structures at higher concentration. The interactive model also highlights that there was a significant interaction between cholesterol and egg lecithin (BC). The negative effect of cholesterol and lecithin interaction could be due to the rigidification of lecithin molecules by cholesterol at higher concentrations, making their bilayer membrane-less leaky and poorly permeable, hindering their drug release characteristics^32,39^. However, the interactive effects of span 60 and lecithin (AC) were observed to have a positive effect on the drug release characteristics. This increased drug release could be due to the formation of a more permeable layer, as the double bonds present in egg lecithin tend to undergo conformational rotation and assemble with non-ionic surfactants (span 60) to form loose junctions^19^.

#### Effect of formulation variables on NIR hyperthermia

**Figure 7:**
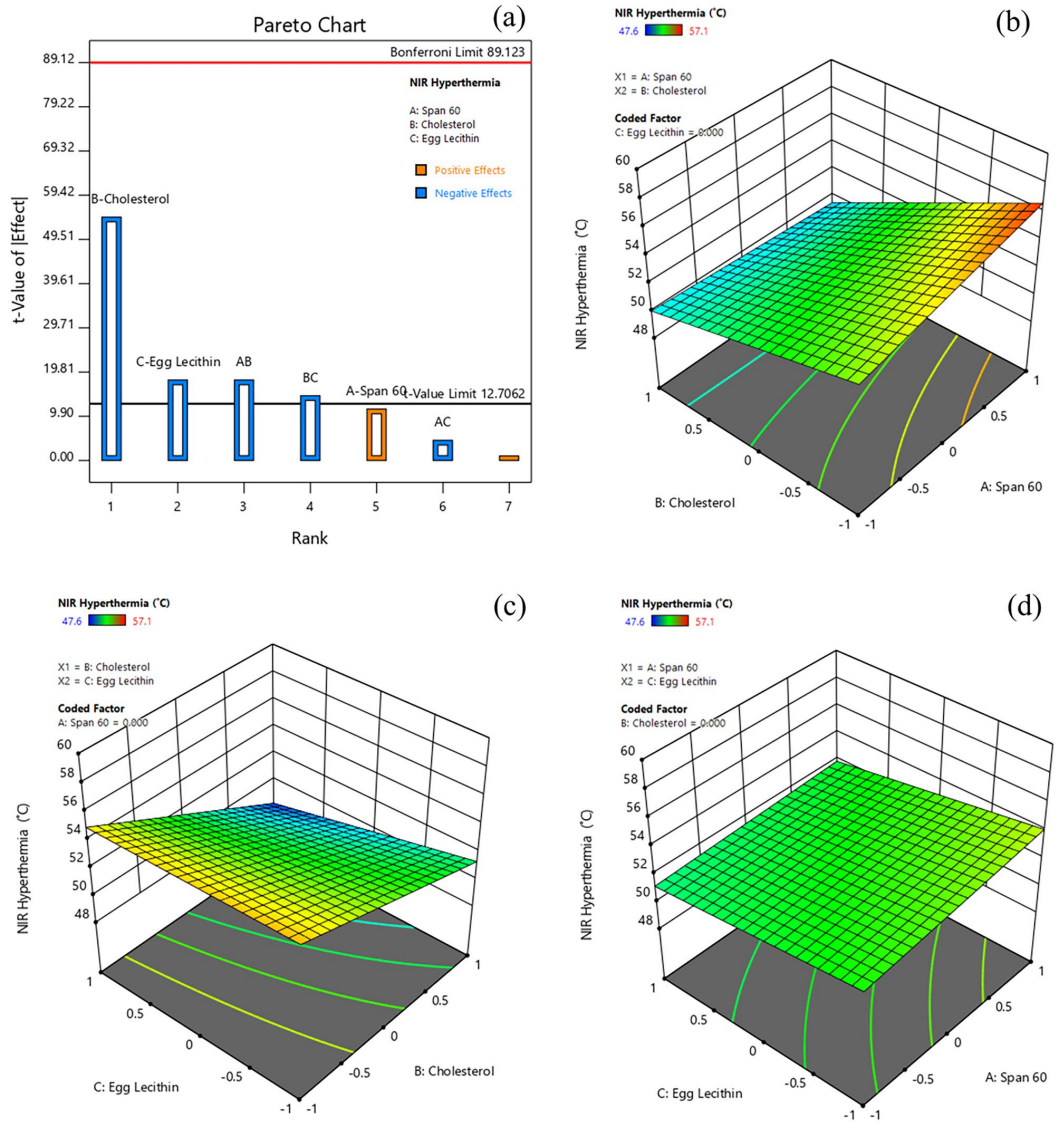
Effect of formulation variables (**a**) Pareto chart of NIR-Hyperthermia, (**b**) Span60*Cholesterol on NIR-Hyperthermia, (**c**) Cholesterol*Egg Lecithin on NIR-Hyperthermia, (**d**) Span60*Egg Lecithin on NIR-Hyperthermia [Design-Expert version 12.0.5.0].

Generally, the heat generation from NIR-based dyes are concentration- dependent, and their heat capacity varies in both free and bound-ICG^15^. NIR-induced Hyperthermia temperature has been considered as one of the critical parameters, as the effect of hyperthermia is dependent on the aggregation concentration of ICG. The hyperthermia temperature of various formulations ranged from 47.6 to 57.1% (Table 3). The Pareto chart and 3D-response surface plots (Design-Expert version 12.0.5.0) indicating the effect of formulation variables on NIR Hyperthermia are presented in Fig. 7a–d.

Table 4 indicates that all factors (B, C, AB, and BC) except A and AC had a significant effect on hyperthermia from Dox-loaded ICG proniosomal gels. The interactive polynomial equation of Y_6_ with coded factors derived from the responses is shown in Eq. (10).

10 $${Y}_{6}=52.45+0.575 A-2.725 B-0.900 C-0.900 AB-0.225 AC-0.725 BC$$

The effect of variables on hyperthermia is comparable to their effect on drug release, indicating their hyperthermia dependence on the availability of the free drug for enhanced NIR hyperthermia effect. Further, the negative effect of cholesterol, egg lecithin, span 60*cholesterol (AB), and cholesterol*egg lecithin (BC) could be correlated with the increased hydrophobicity and decreased permeability of bilayer membrane. However, in order to achieve the clinical benefit, the drug delivery carrier is expected to retard the release of ICG, as free drug undergoes rapid renal elimination and low targeting ability in-vivo. It is noteworthy to design a carrier system with slower drug release characteristics, as the NIR induced hyperthermia efficiency of Dox-loaded ICG proniosomal gel-derived niosomes doesn’t vary profoundly.

### Optimization and validation

**Figure 8:**
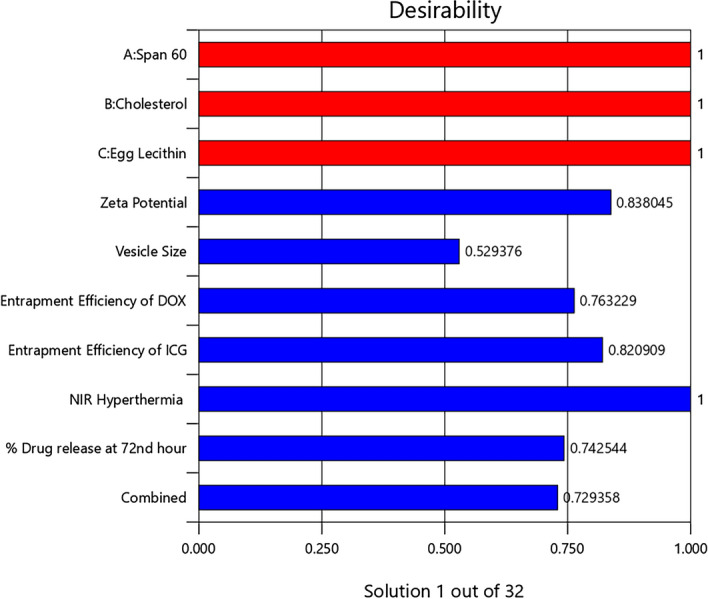
Desirability bar graph for each factor and combination of all responses [Design-Expert version 12.0.5.0].

The optimization of formulation variables is highly desirable to obtain the most stable pharmaceutical product with high-quality robustness. However, achieving multiple desired responses simultaneously is quite impossible, as every response is related to variables in a unique way of its own and interferes with the desirability of other responses. Herein, statistical models such as linear regression and interactive model were used for the understanding of variables influence over the responses. Following numerical optimization analysis, the combination of formulation variables with high desirability has been chosen for validation. The desirability of each factors and the overall desirability of the optimised formulation was shown in the Fig. 8. The predicted and experimental values of responses for chosen Dox-loaded ICG proniosomal gel-derived niosomal (DIP) formulation are highlighted in Table 5.

**Table 5:** Prediction and validation of optimized formulation (DIP).

Factor	Coded value	Actual value (μM)	Response	Predicted value	Experimental value^a^	% Error
Span 60	0.728	13.98	Zeta potential (mV)	− 28.054	− 28.49 ± 1.01	0.037
Cholesterol	− 0.303	6.697	Vesicle size (nm)	202.582	195.36 ± 4.22	3.24
Egg lecithin	− 0.882	6.354	Entrapment efficiency of Dox (%)	78.121	77.24 ± 1.45	0.198
			Entrapment Efficiency of ICG (%)	90.174	91.21 ± 0.91	0.099
			Invitro drug release at 72nd hour (%)	42.994	40.71 ± 3.21	0.438
			NIR Hyperthermia temperature (°C)	54.636	56.17 ± 0.94	0.165

^a^Data represented as the mean ± SD (n = 3).

The estimated errors (%) between the predicted and experimental values were considerably smaller, indicating the desirability of the optimization process. The optimized formulation (DIP) was further investigated to study the morphological and surface characteristics, drug release (%) characteristics and release kinetics, and in-vitro cytotoxicity studies.

### Vesicle morphology

**Figure 9:**
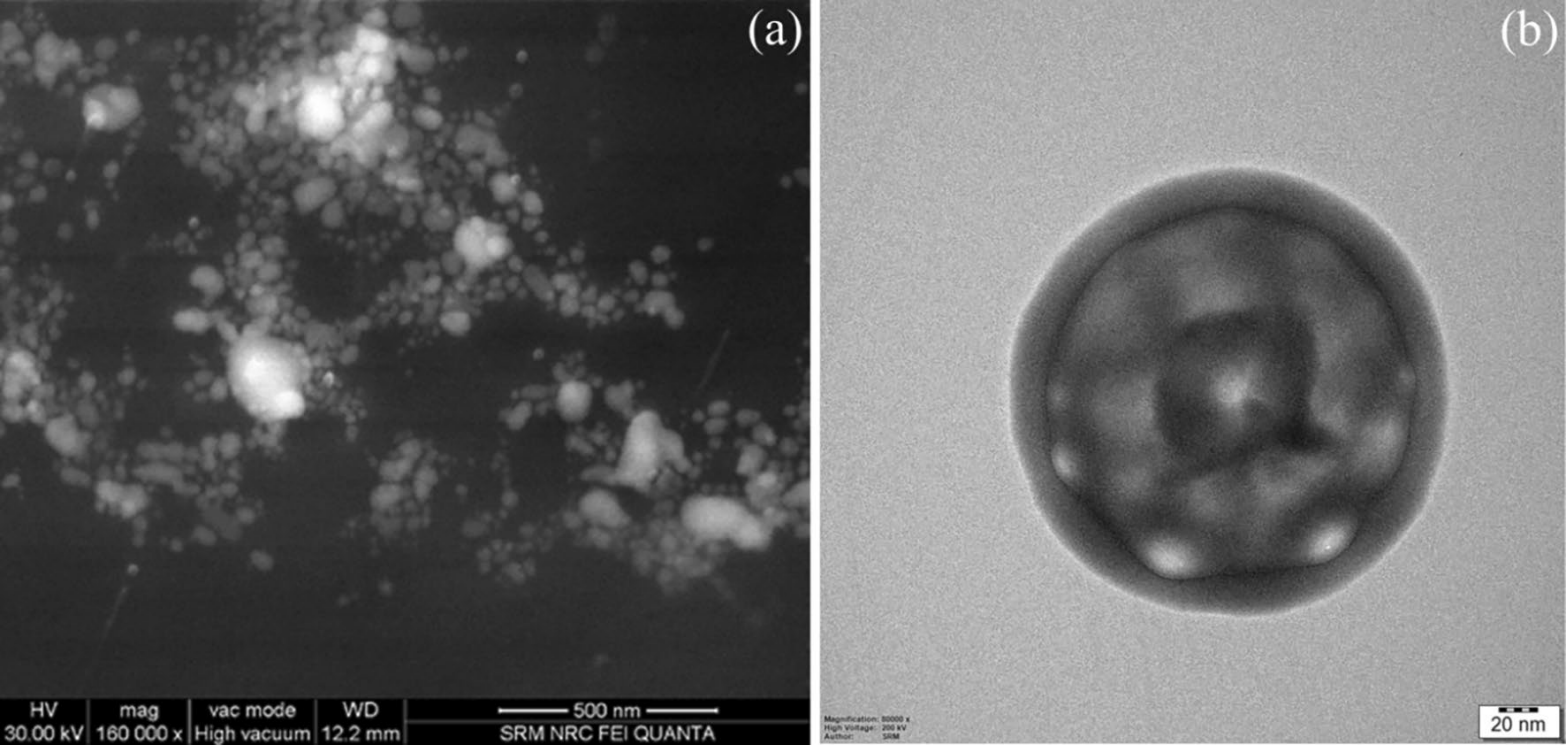
Vesicle morphology, (**a**) FESEM image and (**b**) TEM image of DIP.

FESEM and TEM images of DIP are depicted in Fig. 9a and b. FESEM images of lyophilized DIP revealed spherical and slightly aggregated proniosomes, wherein the aggregation of vesicles could be aroused during lyophillization or dropcasting process of sample preparation. FESEM images also visualises some bright vesicles at certain regions due to charging effect. This charging effect could be due to the presence of high non-ionic span 60 contents in these regions. A similar effect was observed in earlier studies using span 60 as a primary carrier^40^. Further, the TEM image taken for DIP proniosomal gel-derived niosomes revealed a multi-lamellar spherical vesicle with core and bilayer structure, indicating the formation of niosomes upon dispersion with aqueous media.

### Zeta potential and vesicle size

**Figure 10:**
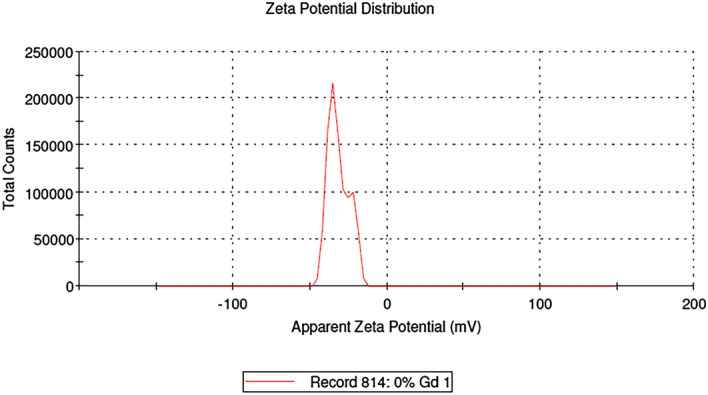
Zeta potential of DIP proniosomal gel-derived niosomes.

**Figure 11:**
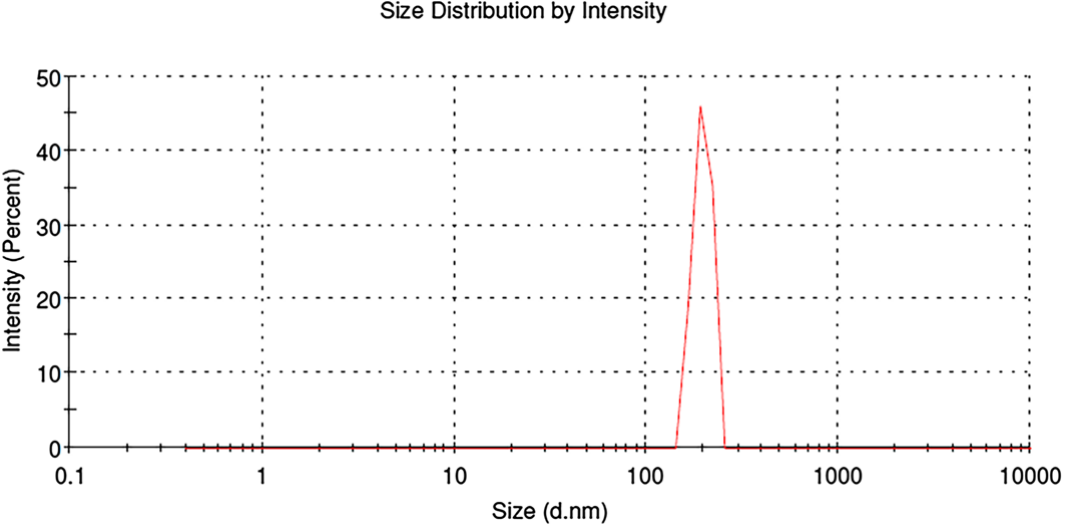
Vesicle size of DIP proniosomal gel-derived niosomes.

The average zeta potential (Fig. 10) of DIP proniosomal-derived niosomes determined using the ELS technique was found to be − 28.49 ± 7.29 mV. The average vesicle size (Fig. 11) of DIP proniosomal-derived niosomes determined using the DLS technique was found to be 195.36 ± 44.11 nm, and their PDI value of 0.051indicates the monodisperse nature of DPI proniosomal-derived niosomes.

The wide range of zeta potential could be related to the varying vesicle size observed under FESEM and DLS techniques. The PDI value below 0.1 indicates that prepared proniosomal-derived niosomes are highly stabilized and monodisperse, which could be due to both steric and electrostatic stabilization of niosomes exhibited by the larger hydrophobic chain length and dominant phosphate moieties of egg lecithin in the formulation^29^.

### NIR hyperthermia measurement

**Figure 12:**
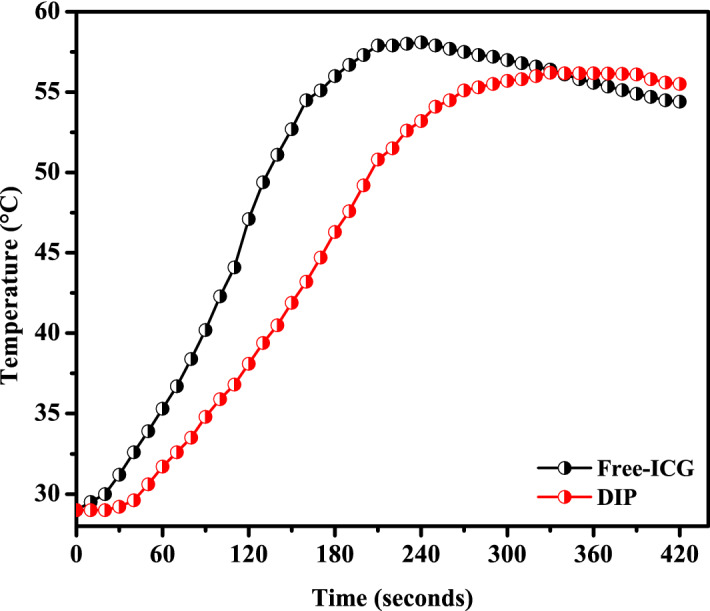
NIR Hyperthermia of Free-ICG and DIP proniosomal gel-derived niosomes.

The heat generation capability of the free and bound form (DIP) of Indocyanine green (⁓ 10 µg/mL) dispersed in phosphate buffer pH 7.4 was recorded and depicted in Fig. 12 generated using Origin(Pro) 8.5^41^. The NIR hyperthermia measurement was performed by irradiating the sample at 808 nm using a UV–Vis-NIR spectrophotometer and recording the change in temperature of the test sample using a Neoptix T1™ optical temperature sensor. The free drug exhibited an increased rate of NIR heating and achieved a heating efficiency of about 58.1 °C at 240 s. Whereas the formulation DIP exhibited a slightly slower rate of heating than free ICG and achieved a maximum of about 56.2 °C at 330 s. It was also observed that the temperature-induced in both free ICG and bound ICG samples decays over time. This decay in temperature could be due to the degradation of ICG or loss of fluorescence activity of ICG at increased temperature and aqueous environment. It was also observed that bound ICG exhibited a lower degradation rate than free ICG, which indicates that degradation rate due to the aqueous vehicle is minimal inbound ICG, as it is protected by the hydrophobic environment of niosomal dispersion^14,15,42^.

### Invitro drug release and drug release kinetics

**Figure 13:**
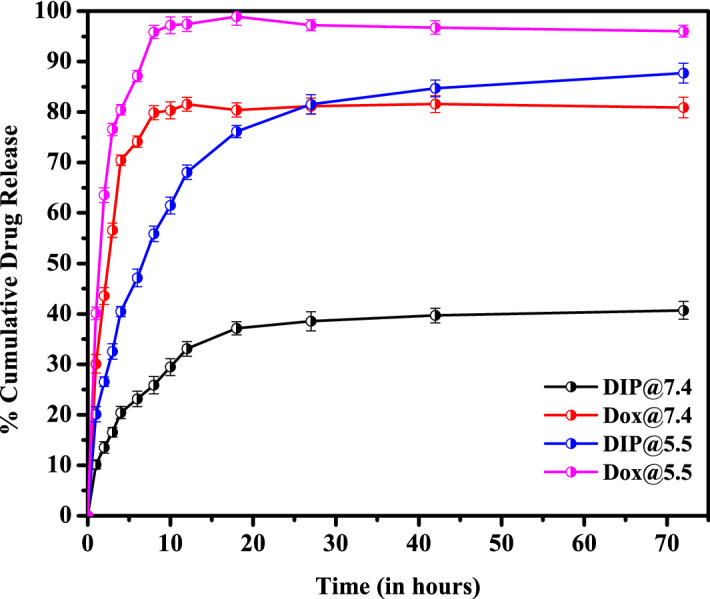
Percentage drug release of Doxorubicin hydrochloride and DIP proniosomal gel-derived niosomes [Origin(Pro) 8.5].

The in-vitro drug release studies (Fig. 13) of pure drug doxorubicin performed under phosphate buffer pH 7.4 and acetate buffer pH 5.5 showed that the drug release is higher and complete in slightly acidic medium than the physiological pH. This type of release pattern is mainly due to the limited solubility of drug doxorubicin hydrochloride in the physiological pH^43^. The study aimed to restrict the release of drug in the physiological medium, so as to reduce the adverse effects on non-targeted tissues invivo. The in-vitro drug release studies of DIP proniosomal gel-derived niosomes revealed restricted drug release in the phosphate buffer pH 7.4, while at pH 5.5, there is a significant enhancement in the release of drug doxorubicin, due to its enhanced solubility in slightly acidic pH conditions. In both pH conditions, a controlled release of doxorubicin was observed. At pH 7.4, it was observed that about 37.14% of doxorubicin was released at the first phase within 18 h of the start of in-vitro drug release study and about 40.71% of doxorubicin was released at the end of the 72nd hour. In contrast, at pH 5.5, about 76.14 ± 1.32% and 87.71 ± 1.97% of doxorubicin was released at 18th hour and 72nd hour respectively. The release pattern highlights that the formulation DIP possesses a biphasic release of drug with loading and maintenance dose at both pH conditions. Further, the release study also revealed that the membrane is not a rate limiting factor for the drug release. The in-vitro cumulative percentage drug release of doxorubicin from DIP proniosomal gel-derived niosome at pH 5.5 and 7.4 was fitted into various mathematical pharmacokinetic models (Supplementary Information), and the goodness of fit was indicated by regression coefficients (Table 6). Among the various models, the Weibull model represented a regression value close to 1, indicating the heterogeneous matrices of DIP proniosomal gel-derived niosomes with high and low diffusivity regions. Also, the β value obtained from the Weibull model was found to be higher than 1, indicating the complex mechanics involved in the drug transport^44,45^.

**Table 6:** Drug release kinetics of Dox-loaded ICG Proniosomes.

Sample ID	Release kinetic models								
	First order	Zero order	Korsemeyer Peppas		Higuchi diffusion	Hixson Crowel	Noyes-Whiney	Weibull	
	R^2^	R^2^	R^2^	N	R^2^	R^2^	R^2^	R^2^	β
DIP@7.4	0.5946	0.5439	0.9227	0.348	0.8147	0.615	0.9436	0.9991	1.147
DIP@5.5	0.7857	0.5755	0.9302	0.3713	0.8388	0.7397	0.7986	0.9834	1.5004

### Invitro anti-cancer activity

**Figure 14:**
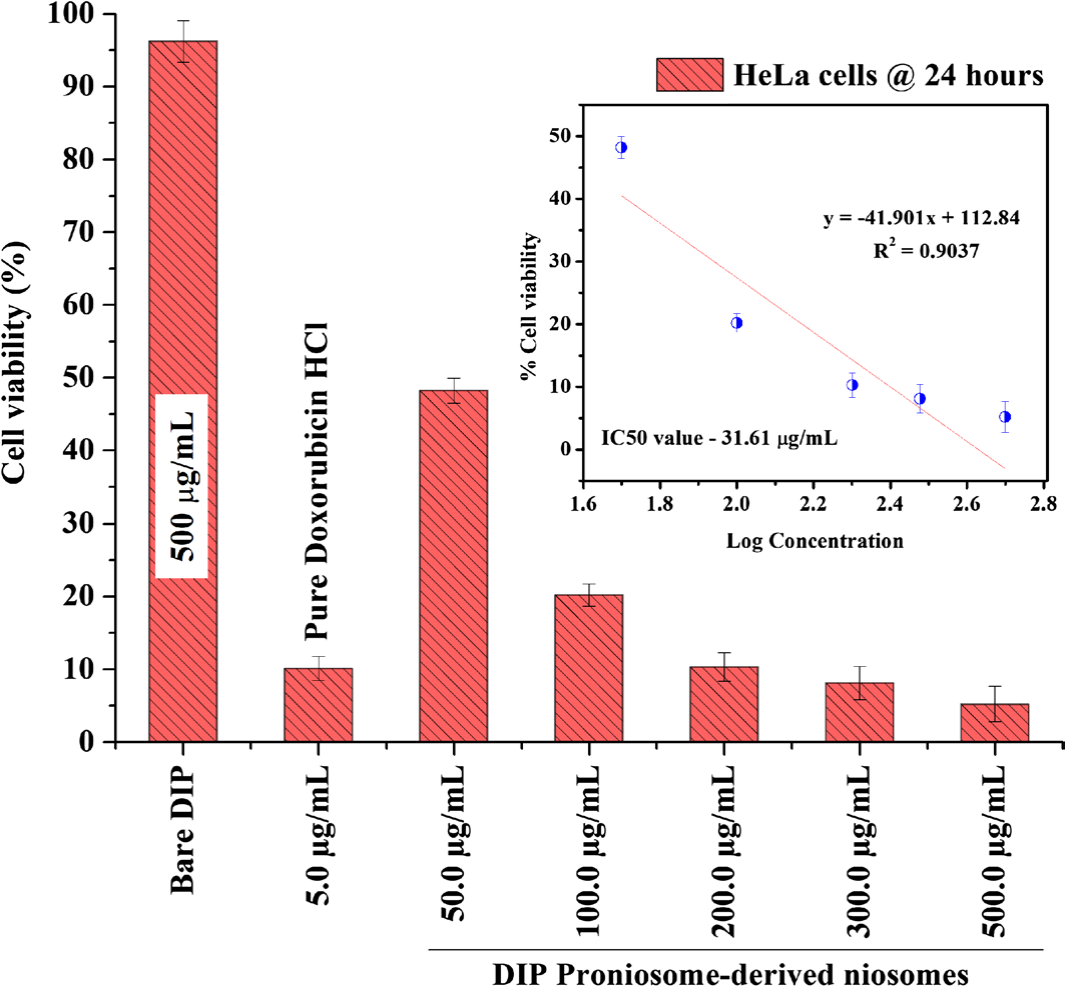
Invitro anticancer activity of DIP proniosomal gel-derived niosomes.

The invitro anti-cancer activity of DIP proniosomal gel-derived niosomes was performed on HeLa cells and the results are reported in Fig. 14 generated using Origin(Pro) 8.5. The treatment of HeLa cells with 500 µg/mL of bare proniosomal gel doesn’t significantly affect their percentage viability. In contrast, the positive control doxorubicin hydrochloride (5 µg/mL) showed a significant reduction in viability of HeLa cells. Further, DIP proniosomal gel-derived niosomes has showed dose dependent toxicity on HeLa cells. The percentage cell viability was found to be 96.23 ± 2.84, 10.12 ± 1.65%. 48.23 ± 1.72%, 20.22 ± 1.50%, 10.31 ± 1.96%, 8.13 ± 2.29%, and 5.23 ± 2.45% after the treatment of 500 µg/mL of bare proniosomal gel, 5 µg/mL of pure doxorubicin hydrochloride, 50 µg/mL, 100 µg/mL, 200 µg/mL_,_ 300 µg/mL and 500 µg/mL of DIP proniosomal gel-derived niosomes respectively at the end of 24th hour. The significant reduction in cell viability describes the in-vitro cytotoxic potential of DIP proniosomal gel-derived niosomes. The IC50 value of DIP proniosomal gel-derived niosomes was found to be 31.61 µg/mL.

## Conclusion

Non-ionic surfactant-based vesicular systems are one of the effective nanocarrier systems that can accommodate multiple cargo molecules with the both hydrophilic and lipophilic profiles. These vesicular systems are generally biocompatible and are not sequestered in the liver. In this study, we have developed a proniosomal gel, as it can offer better stability and reduced tendency to form aggregates during storage. The statistical models revealed that the formulation variables and their interactions played a critical role in the modulation of responses. Following numerical optimization analysis, the combination of formulation variables with high desirability has been chosen and validated. Further, the surface characteristics such as morphology, vesicle size, polydispersity index, and zeta potential revealed the spherical vesicular structure of niosomal dispersion with optimum vesicle size and surface charge. The drug release studies and release kinetics revealed a biphasic release profile of optimized DIP proniosomal gel-derived niosomes with complex drug release kinetics. Overall, the physical stability, high entrapment efficiency, NIR induced hyperthermia potential, and controlled drug release of DIP proniosomal gel-derived niosomes describes their potential to act as a multifunctional drug delivery system towards the management of tumors. In addition, the evaluation of diagnostic potential of such proniosomal gel-derived niosomes shall make them a potential candidate for theranostic applications.

## Supplementary Information

Supplementary Information.

## Data Availability

Data pertaining to this manuscript shall be available from the corresponding author on reasonable request.

## References

[1] Wen H, Jung H, Li X (2015). Drug delivery approaches in addressing clinical pharmacology-related issues: Opportunities and challenges. AAPS J..

[2] Gupta S, Kumar P (2012). Drug delivery using nanocarriers: Indian perspective. Proc. Natl. Acad. Sci. India Sect. B Biol. Sci..

[3] Li Q, Zhou Y, He W, Ren X, Zhang M, Jiang Y, Zhou Z, Luan Y (2021). Platelet-armored nanoplatform to harmonize janus-faced IFN-γ against tumor recurrence and metastasis. J. Control Release.

[4] Zhang M, Qin X, Xu W, Wang Y, Song Y, Garg S, Luan Y (2021). Engineering of a dual-modal phototherapeutic nanoplatform for single NIR laser-triggered tumor therapy. J. Colloid Interface Sci..

[5] Zhou S, Shang Q, Wang N, Li Q, Song A, Luan Y (2020). Rational design of a minimalist nanoplatform to maximize immunotherapeutic efficacy: Four birds with one stone. J. Control Release.

[6] Kazi KM, Mandal AS, Biswas N, Guha A, Chatterjee S, Behera M, Kuotsu K (2010). Niosome: A future of targeted drug delivery systems. J. Adv. Pharm. Technol. Res..

[7] Pattnaik S, Swain K, Singh SP, Sirbaiya AK (2020). Lipid vesicles: Potentials as drug delivery systems. Nanoeng. Biomater. Adv. Drug Deliv..

[8] Akhtar N (2014). Vesicles: A recently developed novel carrier for enhanced topical drug delivery. Curr. Drug Deliv..

[9] Kapoor B, Gupta R, Gulati M, Singh SK, Khursheed R, Gupta M (2019). The why, where, who, how, and what of the vesicular delivery systems. Adv. Colloid Interface Sci..

[10] Yasam VR, Jakki SL, Natarajan J, Kuppusamy G (2013). A review on novel vesicular drug delivery: Proniosomes. Drug Deliv..

[11] Bhavani GD, Veeralakshmi P (2020). Recent advances of non-ionic surfactant-based nano-vesicles (niosomes and proniosomes): A brief review of these in enhancing transdermal delivery of drug. Futur. J. Pharm. Sci..

[12] Muzzalupo R, Tavano L (2015). Niosomal drug delivery for transdermal targeting: Recent advances. Res. Rep. Transderm. Drug Deliv..

[13] Bayindir ZS, Yuksel N (2015). Provesicles as novel drug delivery systems. Curr. Pharm. Biotechnol..

[14] Barnes KD, Shafirstein G, Webber JS, Koonce NA, Harris Z, Griffin RJ (2013). Hyperthermia-enhanced indocyanine green delivery for laser-induced thermal ablation of carcinomas. Int. J. Hyperthermia.

[15] Pham PTT, Le XT, Kim H, Kim HK, Lee ES, Oh KT (2020). Indocyanine green and curcumin co-loaded nano-fireball-like albumin nanoparticles based on near-infrared-induced hyperthermia for tumor ablation. Int. J. Nanomed..

[16] Zheng X, Xing D, Zhou F, Wu B, Chen WR (2011). Indocyanine green-containing nanostructure as near infrared dual-functional targeting probes for optical imaging and photothermal therapy. Mol. Pharm..

[17] Rafiyath SM, Rasul M, Lee B, Wei G, Lamba G, Liu D (2012). Comparison of safety and toxicity of liposomal doxorubicin vs conventional anthracyclines: A meta-analysis. Exp. Hematol. Oncol..

[18] Design-Expert, Version 12.0.5.0. Stat-Ease Inc., Minneapolis, MN, USA https://www.statease.com/ (2022).

[19] Shah H, Nair AB, Shah J, Bharadia P, Al-Dhubiab BE (2019). Proniosomal gel for transdermal delivery of lornoxicam: Optimisation using factorial design and invivo evaluation in rats. Daru.

[20] Gaikwad, V.L., Choudhari, P.B., Bhatia, N.M. & Bhatia, M.S. Chapter 2—characterization of pharmaceutical nanocarriers: In vitro and in vivo studies. In *Nanomaterials for Drug Delivery and Therapy* (ed Grumezescu, A.M.) 33–58 (William Andrew Publishing, 2019).

[21] Deng K, Hou Z, Deng X, Yang P, Li C, Lin J (2015). Enhanced antitumour efficacy by 808 nm laser-induced synergistic photothermal and photodynamic therapy based on indocyanine-green-attached W_18_O_49_ Nanostructure. Adv. Func. Mater..

[22] Guo W, Song Y, Song W, Liu Y, Liu Z, Zhang D, Tang Z, Bai O (2020). Co-delivery of doxorubicin and curcumin with polypeptide nanocarrier for synergistic lymphoma therapy. Sci. Rep..

[23] Liu P, De Wulf O, Laru J, Heikkilä T, van Veen B, Kiesvaara J, Hirvonen J, Peltonen L, Laaksonen T (2013). Dissolution studies of poorly soluble drug nanosuspensions in non-sink conditions. AAPS Pharm. Sci. Tech..

[24] Phillips DJ, Pygall SR, Cooper VB, Mann JC (2012). Overcoming sink limitations in dissolution testing: A review of traditional methods and the potential utility of biphasic systems. J. Pharm. Pharmacol..

[25] Pourtalebi Jahromi L, Ghazali M, Ashrafi H, Azadi A (2020). A comparison of models for the analysis of the kinetics of drug release from PLGA-based nanoparticles. Heliyon..

[26] Lee H, Park H, Noh GJ, Lee ES (2018). pH-responsive hyaluronate-anchored extracellular vesicles to promote tumor-targeted drug delivery. Carbohydr. Polym..

[27] Kanwal U, Bukhari NI, Rana NF, Rehman M, Hussain K, Abbas N, Mehimood A, Raza A (2018). Doxorubicin-loaded quarternary ammonium palmitoyl glycol chitosan polymeric nanoformulation: Uptake by cells and organs. Int. J. Nanomed..

[28] Teja SPS, Damodharan N (2018). 2^3^ Full factorial model for particle size optimization of methotrexate loaded chitosan nanocarriers: A design of experiments (DoE) Approach. BioMed. Res. Int..

[29] Mouzouvi CRA, Umerska A, Bigot AK, Saulnier P (2017). Surface active properties of lipid nanocapsules. PLoS ONE.

[30] Essa EA (2010). Effect of formulation and processing variables on the particle size of sorbitan monopalmitate niosomes. Asian J. Pharm..

[31] Shah P, Goodyear B, Haq N, Puri V, Michniak-Kohn B (2020). Evaluations of Quality by Design (QbD) elements impact for developing niosomes as a promising topical drug delivery platform. Pharmaceutics.

[32] Tabandeh H, Mortazavi SA (2013). An investigation into some effective factors on encapsulation efficiency of alpha-tocopherol in MLVs and the release profile from the corresponding liposomal gel. Iran J. Pharm. Res..

[33] Asthana GS, Sharma PK, Asthana A (2016). Invitro and invivo evaluation of niosomal formulation for controlled delivery of clarithromycin. Scientifica.

[34] Bozzuto G, Molinari A (2015). Liposomes as nanomedical devices. Int. J. Nanomed..

[35] Loo CH, Basri M, Ismail R, Lau HLN, Tejo BA, Kanthimathi MS, Hassan HA, Choo YM (2013). Effect of compositions in nanostructured lipid carriers (NLC) on skin hydration and occlusion. Int. J. Nanomed..

[36] Thomas L, Viswanad V (2012). Formulation and optimization of clotrimazole-loaded proniosomal gel using 3^2^ factorial design. Sci. Pharm..

[37] Dehaghi MH, Haeri A, Keshvari H, Abbasian Z, Dadashzadeh S (2017). Dorzolamide loaded niosomal vesicles: Comparison of passive and remote loading methods. Iran J. Pharm. Res..

[38] Varshosaz J, Andalib S, Tabbakhian M, Ebrahimzadeh N (2013). Development of lecithin nanoemulsion based organogels for permeation enhancement of metoprolol through rat skin. J. Nanomater..

[39] Yeo LK, Olusanya TOB, Chaw CS, Elkordy AA (2018). Brief effect of a small hydrophobic drug (Cinnarizine) on the physicochemical characterisation of niosomes produced by thin-film hydration and microfluidic methods. Pharmaceutics.

[40] Mady O (2017). Span 60 as a microsphere matrix: Preparation and invitro characterization of novel ibuprofen-span 60 microspheres. J. Surfactants Deterg..

[41] Origin(Pro) 8.5, OriginLab Corporation, Northampton, MA, USA https://www.originlab.com/ (2022).

[42] Saxena V, Sadoqi M, Shao J (2003). Degradation kinetics of indocyanine green in aqueous solution. J. Pharm. Sci..

[43] Sun DD, Wen H, Taylor LS (2016). Non-sink dissolution conditions for predicting product quality and in vivo performance of supersaturating drug delivery systems. J. Pharm. Sci..

[44] Preda IA, Mircioiu I, Mircioiu C, Corlan G, Pahomi G, Prasacu I, Anuta V (2012). Research concerning the development of a biorelevant dissolution test for formulations containing Norfloxacin. I. Modelling of invitro release kinetics. Farmacia.

[45] Mircioiu C, Voicu V, Anuta V, Tudose A, Celia C, Paolino D, Fresta M, Sandulovici R, Mircioiu I (2019). Mathematical modelling of release kinetics from supramolecular drug delivery systems. Pharmaceutics..

