# Supplementary material for: Design development and optimisation of multifunctional Doxorubicin-loaded Indocynanine Green proniosomal gel derived niosomes for tumour management
Source: Sci Rep. 2023 Jan 30;13:1697. doi: 10.1038/s41598-023-28891-8 (PMC9886914; doi:10.1038/s41598-023-28891-8)
Supplement: Supplementary file 1 — Supplementary Information. [file 41598_2023_28891_MOESM1_ESM.pdf]

SUPPLEMENTARY FILES

The interactive effects and model diagnostic plots of formulation variables on responses

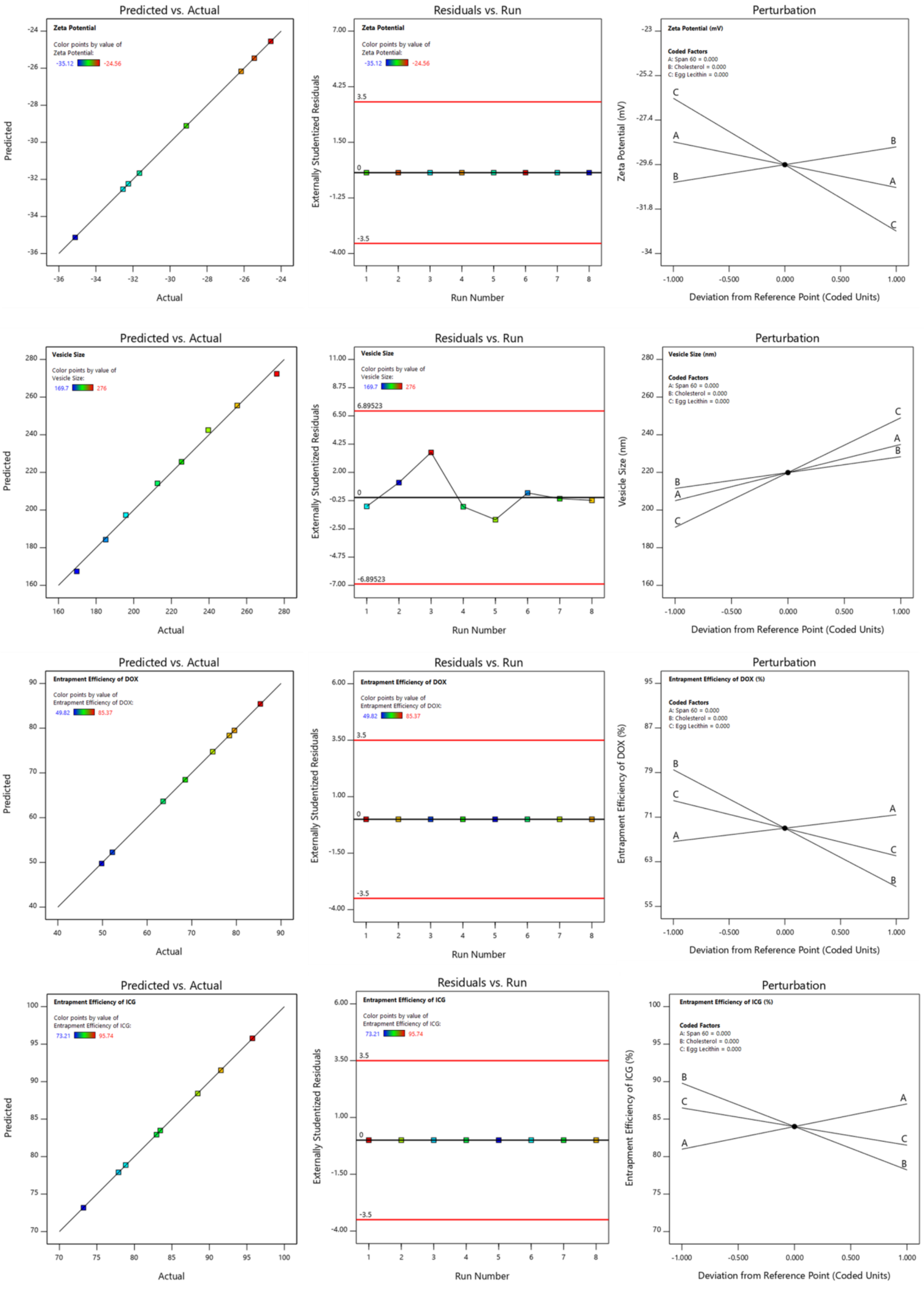

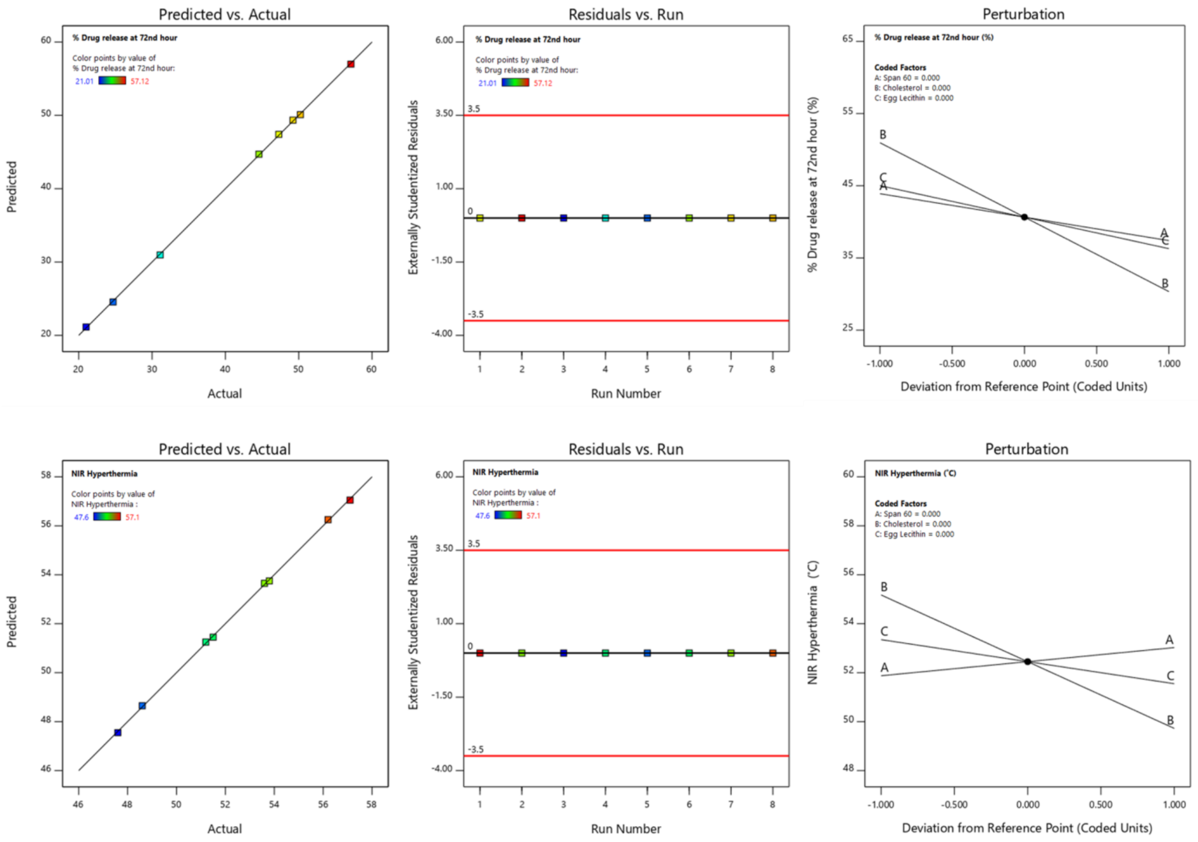
Predicted vs. Actual
% Drug release at 72nd hour
Color points by value of
% Drug release at 72nd hour:
21.01
57.12
Predicted
Actual
Residuals vs. Run
% Drug release at 72nd hour
Color points by value of
% Drug release at 72nd hour:
21.01
57.12
Externally Studentized Residuals
Run Number
Perturbation
% Drug release at 72nd hour (%)
Coded Factors
A: Span 60 = 0.000
B: Cholesterol = 0.000
C: Egg Lecithin = 0.000
% Drug release at 72nd hour (%)
Deviation from Reference Point (Coded Units)
Predicted vs. Actual
NIR Hyperthermia
Color points by value of
NIR Hyperthermia :
47.6
57.1
Predicted
Actual
Residuals vs. Run
NIR Hyperthermia
Color points by value of
NIR Hyperthermia :
47.6
57.1
Externally Studentized Residuals
Run Number
Perturbation
NIR Hyperthermia (°C)
Coded Factors
A: Span 60 = 0.000
B: Cholesterol = 0.000
C: Egg Lecithin = 0.000
NIR Hyperthermia (°C)
Deviation from Reference Point (Coded Units)

Drug release kinetics of DIP proniosome-derived niosomes

The following figures represent the various kinetic models applied to the drug release pattern obtained DIP proniosome-derived niosomes in phosphate buffer pH 7.4

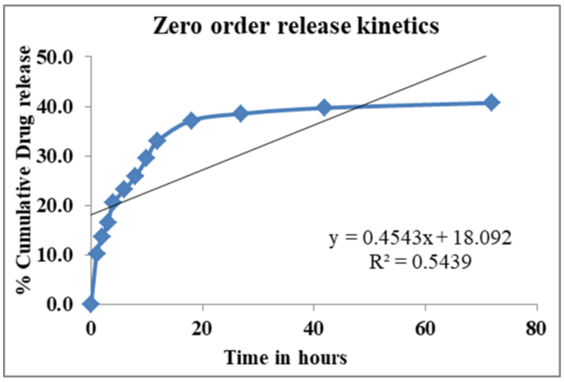

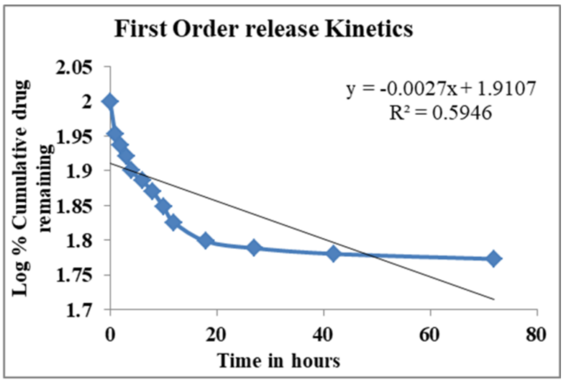

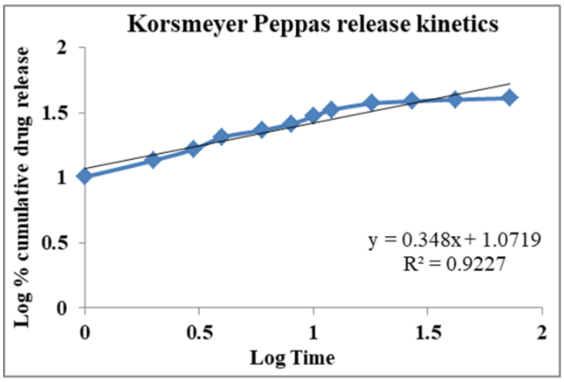

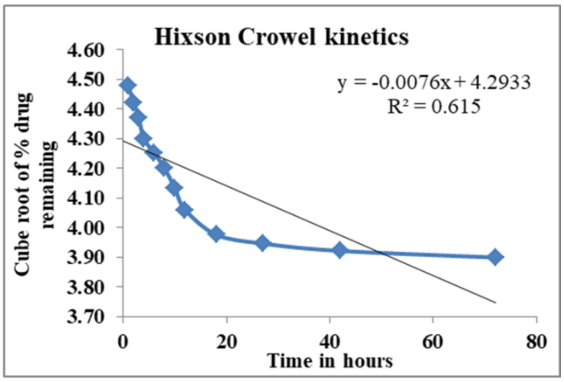

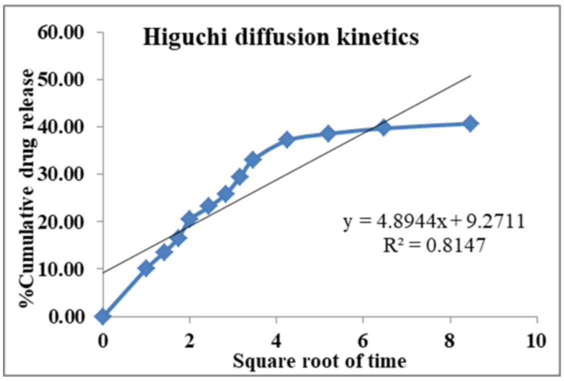

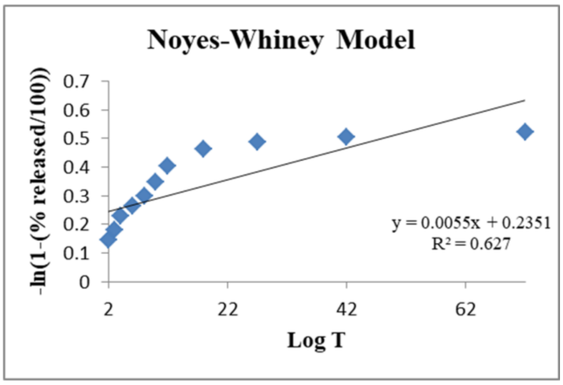

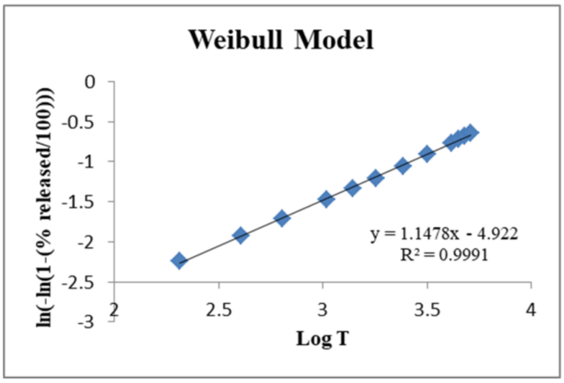

The following figures represent the various kinetic models applied to the drug release pattern obtained DIP proniosome-derived niosomes in phosphate buffer pH 5.5.

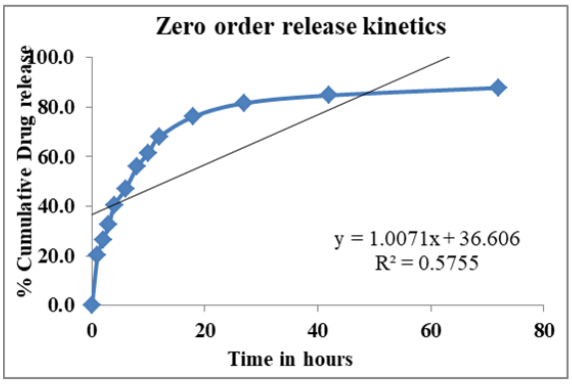

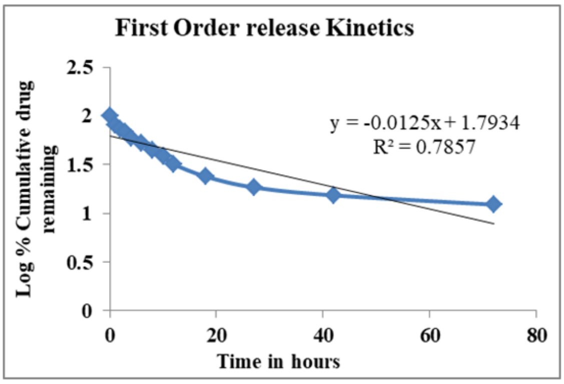

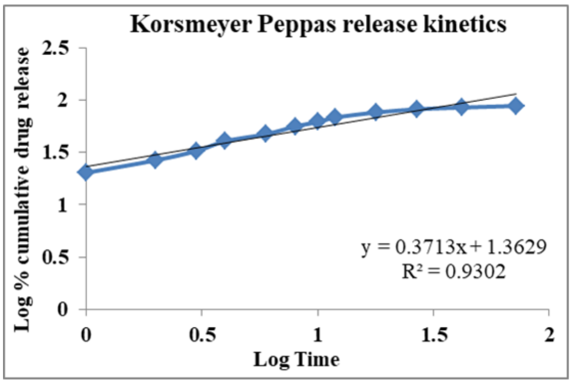

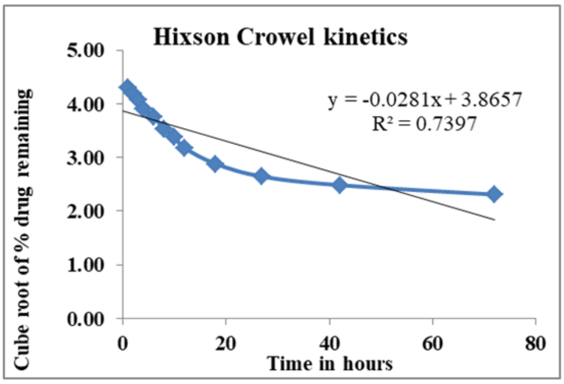

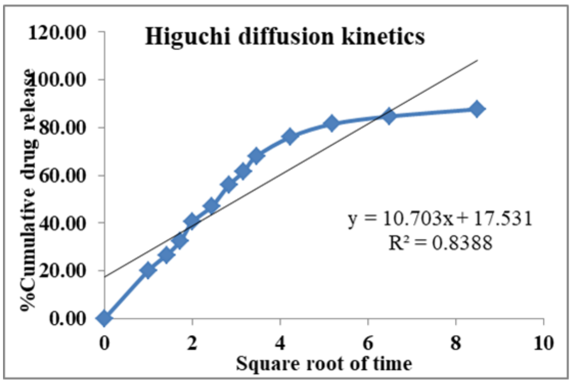

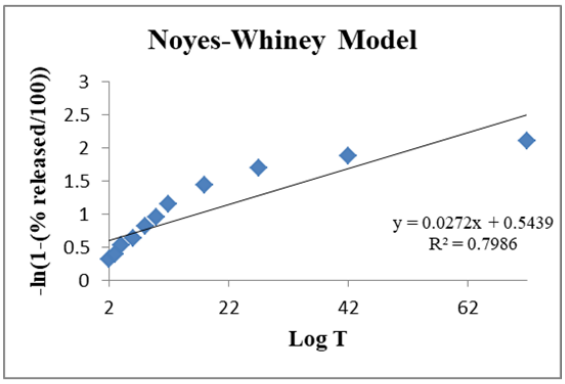

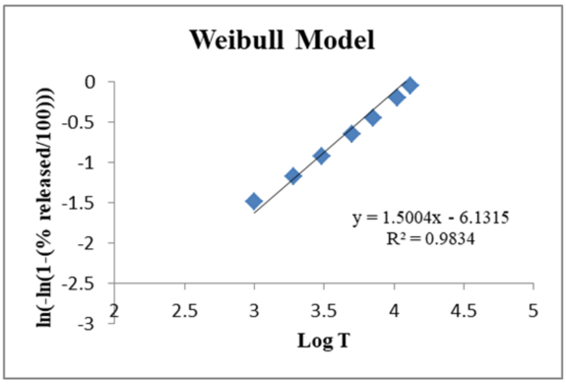

Stability studies of DIP proniosomal-derived niosomes at room temperature

The stability of the prepared DIP proniosomal-derived niosomes was determined from the Entrapment efficiency (%), Zeta potential (mV) and Vesicle size (nm) recorded at initial (0th month) and 8th month.

**Time in months**	**Entrapment Efficiency (%)**		**Zeta Potential (mV)**	**Vesicle Size (nm)**
	**Doxorubicin**	**Indocyanine Green**		
0	77.24 ± 1.45	91.21 ± 0.91	-28.49 ± 1.01	195.36 ± 4.22
8	76.01 ± 1.81	89.97 ± 1.66	-27.13 ± 1.78	205.12 ± 8.09
**% deviation**	1.59 %	1.35 %	4.77 %	-4.99 %

Data represented as the mean±SD (n=3)

Though there is a substantial change in entrapment efficiency, zeta potential and vesicle size of the DIP proniosomal-derived niosomes recorded at 8th month compared to the initial values, the values lies within the desirable range (± 5%).
